# The economical lifestyle of CPR bacteria in groundwater allows little preference for environmental drivers

**DOI:** 10.1186/s40793-021-00395-w

**Published:** 2021-12-14

**Authors:** Narendrakumar M. Chaudhari, Will A. Overholt, Perla Abigail Figueroa-Gonzalez, Martin Taubert, Till L. V. Bornemann, Alexander J. Probst, Martin Hölzer, Manja Marz, Kirsten Küsel

**Affiliations:** 1 Aquatic Geomicrobiology, Institute of Biodiversity, Friedrich Schiller University, Jena, Germany; 2 German Center for Integrative Biodiversity Research (iDiv) Halle-Jena-Leipzig, Leipzig, Germany; 3 Department for Chemistry, Environmental Microbiology and Biotechnology, Group for Aquatic Microbial Ecology (GAME), University Duisburg-Essen, Essen, Germany; 4 RNA Bioinformatics and High Throughput Analysis, Friedrich Schiller University, Jena, Germany; 5 European Virus Bioinformatics Center, Friedrich Schiller University, Jena, Germany; 6 FLI Leibniz Institute for Age Research, Jena, Germany; 7 Present Address: Methodology and Research Infrastructure, MF1 Bioinformatics, Robert Koch Institute, Berlin, Germany

**Keywords:** Candidate phyla radiation (CPR), *Cand.* Patescibacteria, Economic lifestyle, Metagenomics, Microbial ecology

## Abstract

**Background:**

The highly diverse *Cand*. Patescibacteria are predicted to have minimal biosynthetic and metabolic pathways, which hinders understanding of how their populations differentiate in response to environmental drivers or host organisms. Their mechanisms employed to cope with oxidative stress are largely unknown. Here, we utilized genome-resolved metagenomics to investigate the adaptive genome repertoire of Patescibacteria in oxic and anoxic groundwaters, and to infer putative host ranges.

**Results:**

Within six groundwater wells, *Cand*. Patescibacteria was the most dominant (up to 79%) super-phylum across 32 metagenomes sequenced from DNA retained on 0.2 and 0.1 µm filters after sequential filtration. Of the reconstructed 1275 metagenome-assembled genomes (MAGs), 291 high-quality MAGs were classified as *Cand*. Patescibacteria. *Cand*. Paceibacteria and *Cand*. Microgenomates were enriched exclusively in the 0.1 µm fractions, whereas candidate division ABY1 and *Cand*. Gracilibacteria were enriched in the 0.2 µm fractions. On average, Patescibacteria enriched in the smaller 0.1 µm filter fractions had 22% smaller genomes, 13.4% lower replication measures, higher proportion of rod-shape determining proteins, and of genomic features suggesting type IV pili mediated cell–cell attachments. Near-surface wells harbored Patescibacteria with higher replication rates than anoxic downstream wells characterized by longer water residence time. Except prevalence of superoxide dismutase genes in Patescibacteria MAGs enriched in oxic groundwaters (83%), no major metabolic or phylogenetic differences were observed. The most abundant Patescibacteria MAG in oxic groundwater encoded a nitrate transporter, nitrite reductase, and F-type ATPase, suggesting an alternative energy conservation mechanism. Patescibacteria consistently co-occurred with one another or with members of phyla Nanoarchaeota, Bacteroidota, Nitrospirota, and Omnitrophota. Among the MAGs enriched in 0.2 µm fractions,, only 8% Patescibacteria showed highly significant one-to-one correlation, mostly with Omnitrophota. Motility and transport related genes in certain Patescibacteria were highly similar to genes from other phyla (Omnitrophota, Proteobacteria and Nanoarchaeota).

**Conclusion:**

Other than genes to cope with oxidative stress, we found little genomic evidence for niche adaptation of Patescibacteria to oxic or anoxic groundwaters. Given that we could detect specific host preference only for a few MAGs, we speculate that the majority of Patescibacteria is able to attach multiple hosts just long enough to loot or exchange supplies.

**Supplementary Information:**

The online version contains supplementary material available at 10.1186/s40793-021-00395-w.

## Background

Metagenomic sequencing of diverse environments has enabled the recovery of genomic information from a vast majority of uncultivated microbial dark matter, significantly expanding the tree of life. *Cand.* Patescibacteria is a superphylum also known as the Candidate Phyla Radiation (CPR) that constitutes a major portion of this expanded tree of life [1]. Patescibacteria, initially recovered from groundwater and aquatic sediments [2, 3], are now shown to inhabit a broad range of surface and subsurface habitats, such as marine water, freshwater, freshwater beach sands [4] hydrothermal vents [5], cold-water geyser [6, 7], plant rhizosphere [8], alpine permafrost [9], permafrost thaw ponds [10], and many more habitats [11] including the human oral cavity [12–14]. Nevertheless, they dominate some groundwater environments [15–18], thermokarst lakes [19] and hypersaline soda lake sediments [20] where they comprise 20–70% of the total microbial community.

Patescibacteria have small genomes characterized by predicted minimal biosynthetic and metabolic pathways, and are reported to have an anaerobic, fermentative lifestyle [21, 22]. These traits may be responsible for their high abundance in nutrient-limited groundwater habitats, which are mainly anoxic. Interestingly, oxic surface soils are a major source of CPR bacteria inhabiting modern groundwater (stored within last 50 years) [23], as these organisms are easily mobilized into soil seepage water [17, 24], Though there are specific examples of CPR bacteria (Saccharibacteria) coping with oxidative stress in oxic soil environments [25, 26], their metabolic traits to cope with oxidative stress within groundwater environments are largely unknown. Divergent trends in the preference for several hydrochemical parameters or specific host preferences seem to result in the differentiation of CPR bacteria in groundwater [17]. Similarly, little species-level overlap of metagenome-assembled genomes (MAGs) across varying groundwater sites suggests that CPR communities differ based on specific environmental factors including host populations [18].

Most Patescibacteria cells are estimated to have ultra-small diameters ranging from 0.1 µm to 0.3 µm [11, 15, 21] with few exceptions like Saccharimonadia (candidate division TM7) that may be as large as 0.7 µm in diameter [27]. Small cell sizes of Patescibacteria accompanied by reduced genomes [3, 21, 22] suggest host-associated lifestyles. Indeed, specific studies on Patescibacteria isolates along with co-culture and microscopic analyses provided evidence of their symbiotic associations with other organisms e.g. with *Paramecium bursaria*, a ciliated protist in freshwater [28], or with Actinobacteria (*Actinomyces odontolyticus*, *Propionibacterium propionicus*, *Schaalia meyeri*) in the human oral cavity [12, 29–31]. Similarly, CPR bacteria attach as episymbionts to putative bacterial hosts through pilin-like appendages in pristine groundwater [18]. In some cases, parasitic relationships of Patescibacteria with other hosts were also identified [32, 33].

In contrast, single cell genomic and biophysical observations from 46 globally distributed groundwater sites did not support the prevailing view that Patescibacteria are dominated by symbionts [11]. The authors suggest that their unusual genomic features and prevalent auxotrophies may be the result of ancestral, primitive energy metabolism that relies on fermentation. Additionally, genome streamlining in free-living prokaryotes in the open ocean is a known mechanism to reduce functional redundancy and conserve energy [34]. Minimizing energy expenditure and nutrient demands has constituted a selective advantage for *Prochlorococcus* in surface waters where nutrients are scarce at the expense of versatility and competitiveness in changing conditions [35], and the same could be true for CPR bacteria dominating oligotrophic subsurface waters. Thus, there is the need to disentangle which lineages of CPR bacteria are host-dependent and which are free-living, and how much variation in terms of lifestyle, metabolism and gene content exists between those which show a preference for certain geochemical conditions.

In this study, we took advantage of a well-studied modern groundwater system within the Hainich Critical Zone Exploratory (CZE) located in Thuringia, Germany [36], dominated by CPR bacteria, that exhibits large environmental gradients from oxic to anoxic conditions accompanied by different well-specific microbiomes [37]. Using 291 manually curated MAGs we aimed to identify the adaptive genomic repertoire of CPR bacteria. Sequential filtration was performed to gather clues about possible physical association of ultra-small Patescibacteria with larger sized host ranges. We also inferred putative hosts for Patescibacteria based on the co-occurrence patterns with other microorganisms within the transect, especially based on abundances of all the MAGs enriched in the 0.2 µm filter fractions.

## Results

### Patescibacteria dominated Hainich groundwater microbial communities

**Fig. 1:**
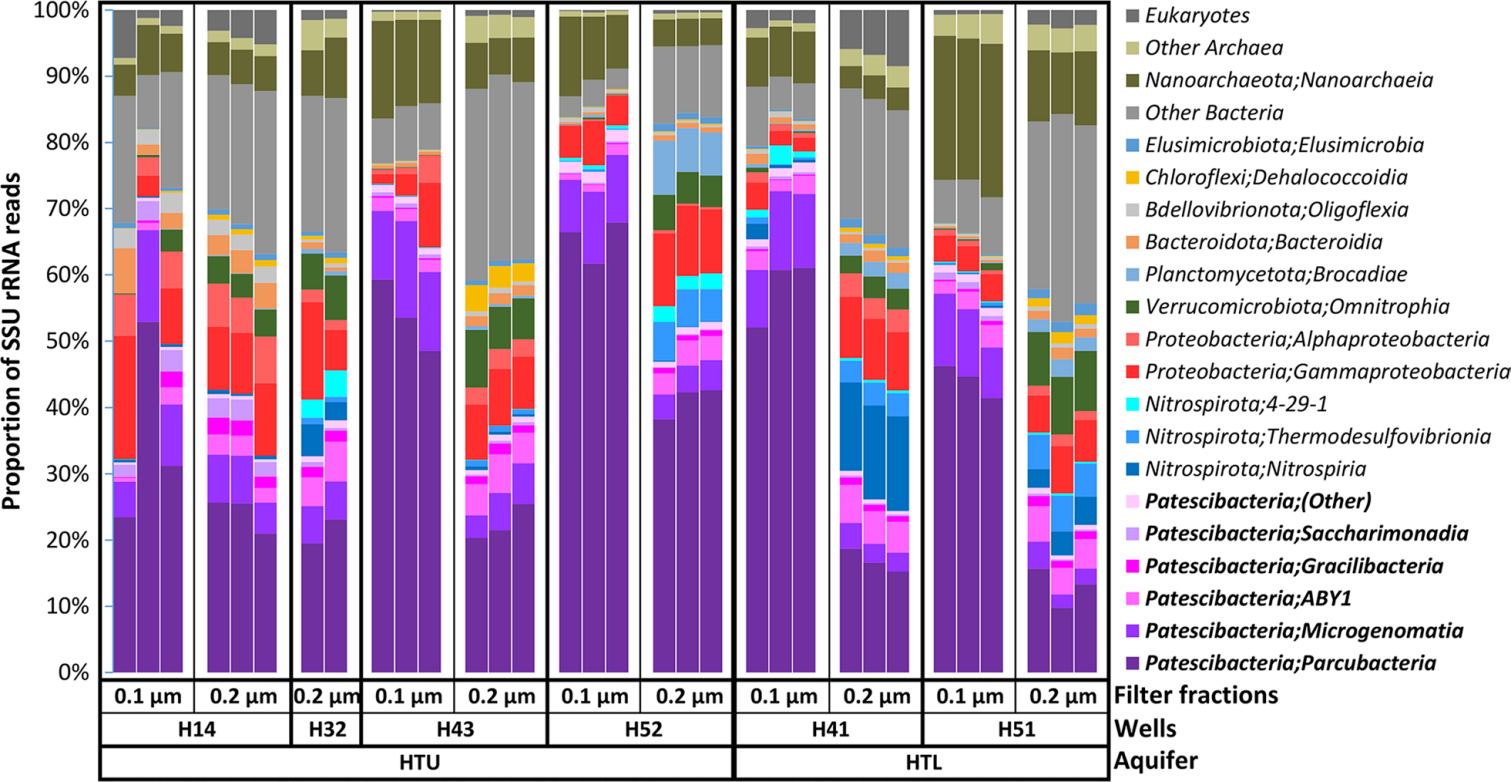
Community composition of the groundwater samples based on metagenomic reads mapped against the SILVA (SSU rRNA Ref NR99) database. Each column represents a metagenomic sample replicate for specified filter fractions from respective wells of the limestone-mudstone strata that host the multi-story upper aquifer assemblage (HTU; wells H14, H32, H43, H52) and the karstified main aquifer (HTL; wells H41, H51)

*Cand*. Patescibacteria dominated the groundwater community across 32 metagenomes obtained from DNA retained on 0.2 µm and 0.1 µm filters after sequential filtration. Based on the proportion of the quality-controlled metagenomic reads mapped to the 16S rRNA database (SILVA SSU rRNA Ref NR99) [38], their average relative abundance in the 0.1 µm filter fractions was 67.6 ± 9.1 (S.D.)% (range 54.1–78.5%) whereas, it was 35.5 ± 8.9% (range 23.1–51.1%) in the 0.2 µm filter fractions.. Their relative abundances were significantly higher (two-proportions z-test, *p* value 1.16e−05) in the 0.1 µm filter fractions than in the 0.2 µm filter fractions. Three major classes within the phylum were detected: *Cand*. Parcubacteria/Paceibacteria with average relative abundance 51.9 ± 9.9%, range 35.5–65.7% in the 0.1 µm filter fractions and 23.1 ± 8.9%, range 13.0–41.3% in the 0.2 µm filter fractions, *Cand*. Microgenomatia with average relative abundance 10.3 ± 0.9%, range 9.6–12% in the 0.1 µm filter fractions and 4.5 ± 1.3%, range 2.8–6.4% in the 0.2 µm filter fractions and candidate division ABY1 with average relative abundance 1.9 ± 0.5%, range 1.1–2.6% in 0.1 µm filter fractions and 4.4 ± 0.9%, range 2.8–5.1% in the 0.2 µm filter fractions (Fig. 1).

**Fig. 2:**
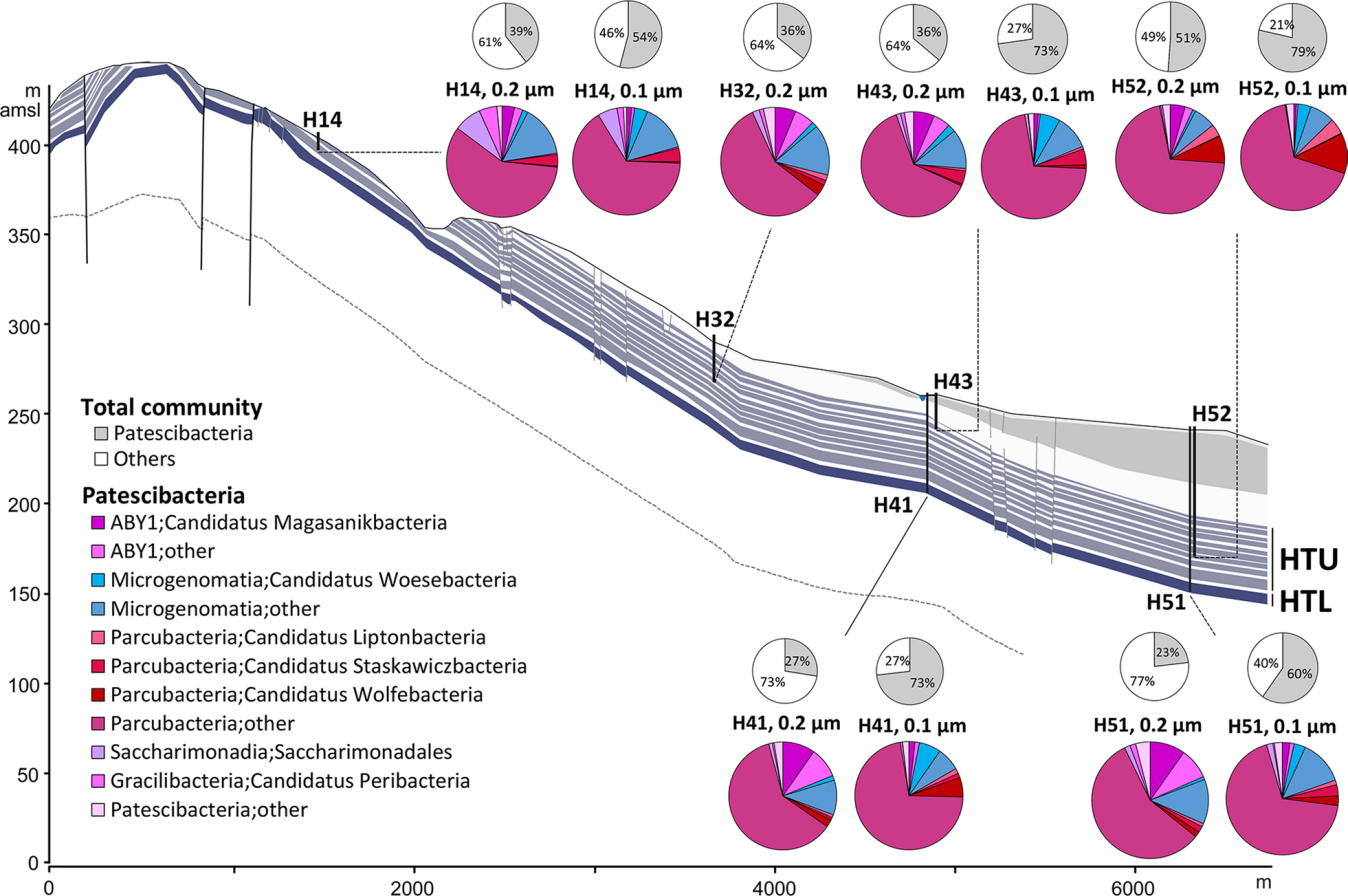
Community composition showing taxonomic preferences of Patescibacteria in wells and filter fractions across the Hainich groundwater transect. The cross section of the studied groundwater transect (from Kohlhepp et al. [48], modified) shows the karstified main aquifer (HTL; wells studied: H41, H51; dark blue line) that is characterized by higher surface-connection to preferential recharge areas and the hanging thin-bedded alternating limestone-mudstone strata that host the multi-story upper aquifer assemblage (HTU; wells studied H14, H32, H43, H52; thin gray-blue lines). Height above mean sea level (amsl), in meters, is shown along the y-axis and length of hillslope is shown in meters along the x-axis. The colored pie charts show percentages of taxa within Patescibacteria at order level. The taxon, *Parcubacteria;other* (all Parcubacteria other than the mentioned Parcubacteria orders merged together) was most abundant among Patescibacteria in all the filter fractions of all the wells. The grey pie charts show the relative percentage of Patescibacteria in the total community

The groundwater transect is characterized by sites (wells) with varying hydrochemical properties (Table 1). Within the detected Patescibacteria, site specific and filter size preferences were observed (Fig. 2). The shallowest well at the top of the hillslope, H14, showed a relatively higher percentage of Saccharimondales compared to other wells. *Candidatus* Staskwiczbacteria showed preference for wells H14 and H43 (characterized by hypoxic/ anoxic environments with low nitrate), and *Candidatus* Wolfebacteria, UBA9983, and *Candidatus* Liptonbacteria for well H52 (characterized by anoxic environment and longest water residence time). *Candidatus* Magasanikbacteria and UBA9983 showed preference for 0.2 µm filter fractions of all the wells, whereas *Candidatus* Woesebacteria was enriched in all the 0.1 µm filter fractions.

**Table 1:** Hydrochemical parameters of groundwater of the Hainich CZE obtained from different wells

Aquifer	Well	pH	Dissolved Oxygen (mg/L)	Ammonium (mg/L)	Nitrate (mg/L)	Sulphate (mg/L)
HTU, slower flow	H14	6.98 ± 0.09 (6.8–7.2)	0.61 ± 0.58 (0.1–2.54)	0.01 ± 0.02 (0–0.06)	1.29 ± 0.21 (0.77–1.52)	26.72 ± 2.16 (23.88–30.2)
	H32	7.31 ± 0.07 (7.2–7.5)	2.23 ± 0.56 (1.31–3.41)	0.01 ± 0.02 (0–0.11)	28.51 ± 8.22 (12.57–40.58)	73.12 ± 5.25 (63.18–91.64)
	H43	7.14 ± 0.07 (7–7.3)	0	0.09 ± 0.06 (0–0.27)	1.55 ± 3.92 (0.01–11.99)	38.52 ± 1.94 (35.15–47.06)
	H52	7.31 ± 0.06 (7.1–7.4)	0	0.41 ± 0.1 (0.13–0.58)	5.35 ± 4.37 (0.07–16.32)	88.66 ± 8.22 (72.81–102.95)
HTL, fast conduit groundwater flow	H41	7.25 ± 0.17 (7.1–8.1)	4.83 ± 1.7 (1.77–8.04)	0.12 ± 0.1 (0–0.33)	10.16 ± 4.41 (2.51–23.33)	91.62 ± 20.76 (59.44–140.48)
	H51	7.15 ± 0.09 (6.9–7.3)	2.73 ± 0.31 (2.21–3.29)	0.04 ± 0.12 (0–0.68)	8.12 ± 3.27 (4.87–21.05)	289.47 ± 19.95 (253.96–337.19)

Four wells access the Hainich transect upper aquifer assemblage (HTU), characterized by slow diffuse flow in slightly fractured, thin aquifer beds; and two wells access the main Hainich transect lower aquifer assemblage (HTL), characterized by fast conduit groundwater flow typical for karstified carbonate rocks (Kohlhepp et al. [48]). The values represent averages with standard deviation from data measured during July 2014–April 2017 [37]. The minimum to maximum ranges are shown in the round brackets

### Dominance of Patescibacteria in Hainich groundwater communities enabled recovery of hundreds of high quality MAGs

**Fig. 3:**
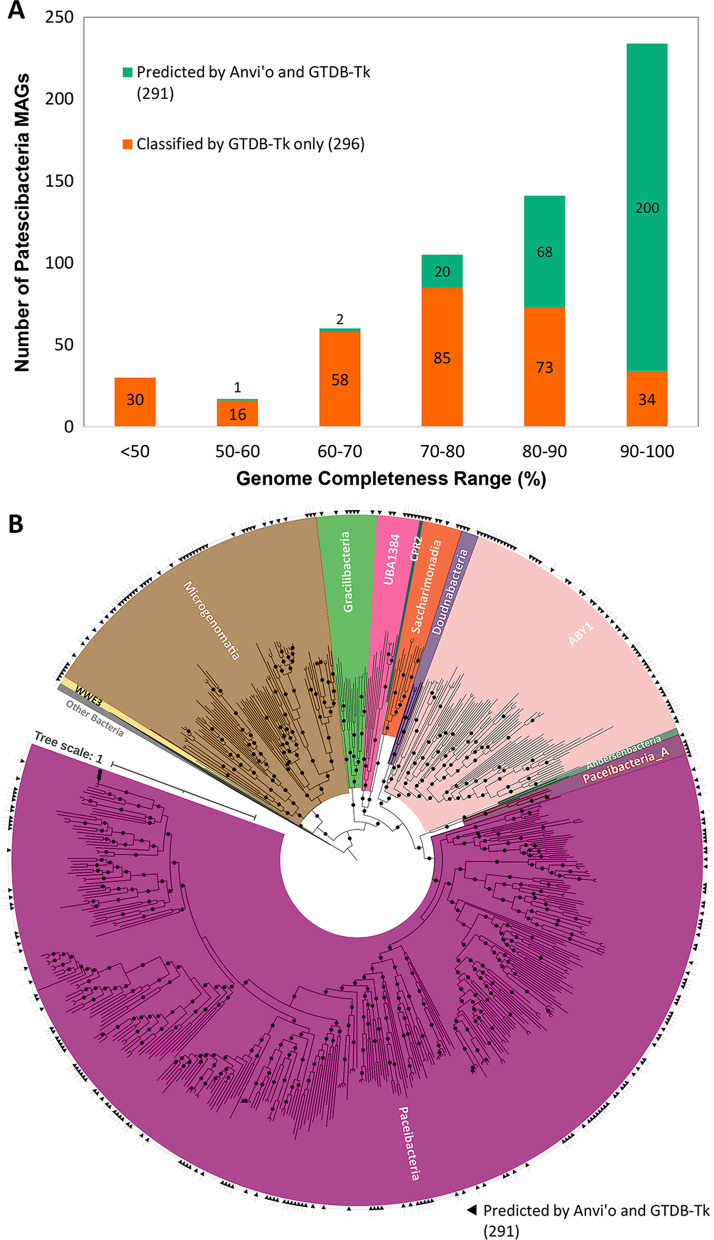
Phylogenetic placement of Patescibacteria MAGs after binning and refinement. **A** Genome completeness distribution of the MAGs classified as Patescibacteria by GTDB-Tk alone (174, orange-colored bars), and by both GTDB-Tk and Anvi’o (291, teal-colored bars). **B** Phylogenetic tree based on 68 core proteins from all bacterial MAGs (1087) using Approximate Maximum Likelihood in FastTree2 with 1000 bootstrap replications. Bacterial taxa other than Patescibacteria were collapsed together and only Patescibacteria are colored as per their taxonomic assignments from GTDB-Tk. The bootstrap values of 0.9 and above are indicated by filled circles. The phylogenetic tree is supplied as Additional file 3

Metagenomic assembly and binning of all individual groundwater samples (n = 32) yielded a total of 1275 non-redundant manually refined MAGs from various bacterial and archaeal species. Among these MAGs, 584 MAGs were classified as *Cand*. Patescibacteria by GTDB-Tk and 291 of them were classified as CPR with high confidence score by a random forest classifier within Anvi’o v6.1 [39, 40], trained with a set of CPR specific single copy genes extracted from previously published CPR genomes [15, 41] (Additional file 1). Most of these 291 MAGs belonged to the classes: *Cand*. Paceibacteria (163 MAGs) followed by candidate division ABY1 (49 MAGs), and *Cand*. Microgenomatia (46 MAGs) (Fig. 3A). The details about all the Patescibacteria MAGs are provided in Additional file 2. The phylogenetic tree constructed from the multiple alignment of 68 core protein sequences confirmed the taxonomic placement of Patescibacteria MAGs (Fig. 3B).

### Differences in the genome sizes of Patescibacteria based on cell size enrichment

We identified 110 Patescibacteria MAGs enriched in the 0.1 µm filter fractions based on their average *rpoB* gene-count-normalized coverage (See Methods) being fivefold higher than in the 0.2 µm filter fractions. Of these, 82 MAGs were further classified as *Cand*. Paceibacteria, and 23 as *Cand*. Microgenomatia. Both classes were absent in the MAGs enriched in 0.2 µm filter fractions. Similarly, 33 Patescibacteria MAGs were enriched fivefold more in the 0.2 µm filter fractions, with 22 of those belonging to the candidate division ABY1, and 5 to *Cand*. Gracilibacteria. Again, none of the genomes classified in these two classes were enriched in the 0.1 µm filter fractions.

**Fig. 4:**
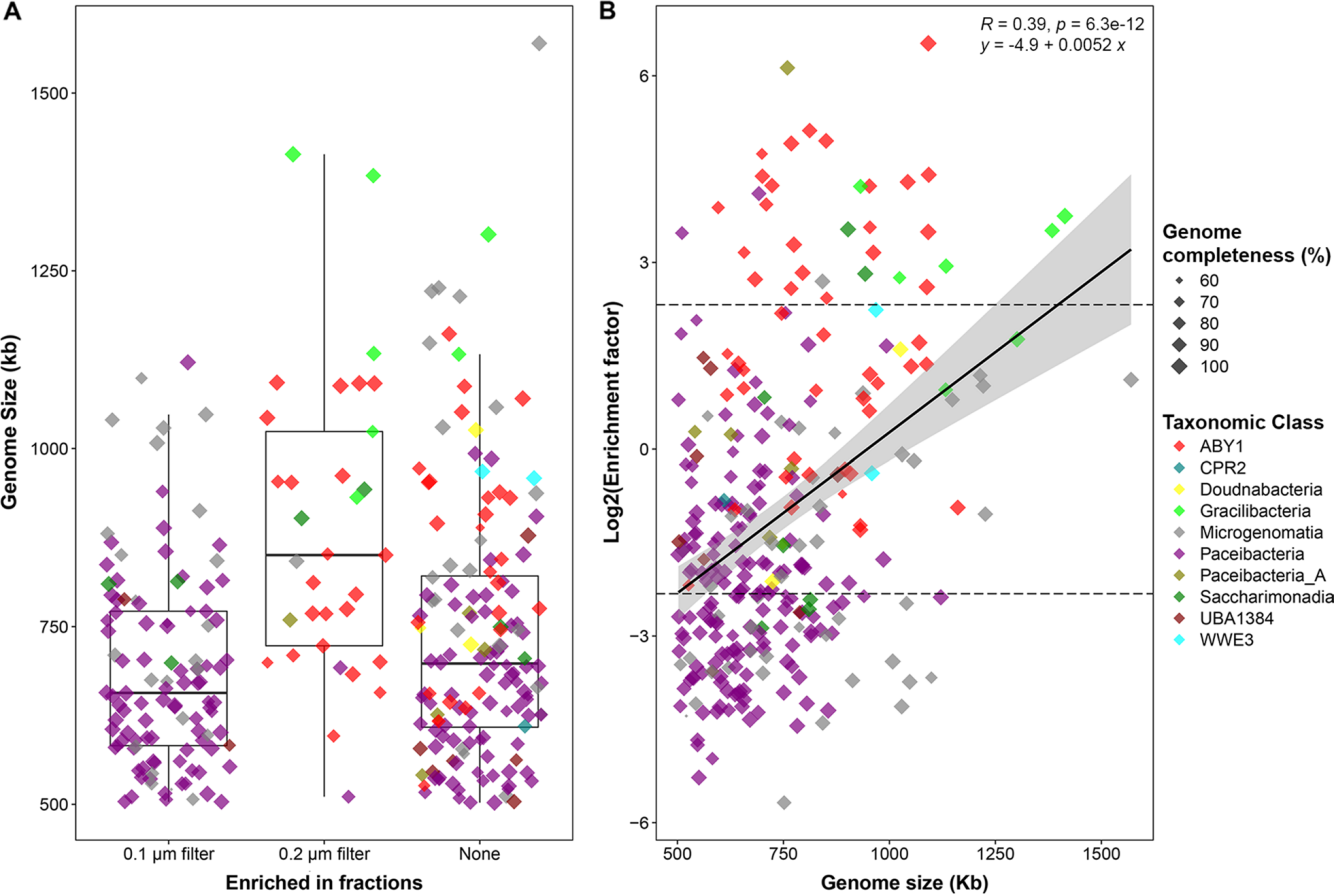
Distribution of genome sizes of Patescibacteria MAGs enriched in 0.1 µm and 0.2 µm filter fractions. **A** For all 291 high-quality Patescibacteria MAGs, the ratio of average normalized genome coverage in 0.1 µm filter fractions to 0.2 µm filter fractions from metagenomes was used to form three groups: ‘0.1 µm filter’—MAGs where this ratio was at least 5, ‘0.2 µm filter’—MAGs where this ratio was 1/5 or less, and ‘None’—MAGs other than first two groups. The mean genome sizes were significantly different (Kruskal–Wallis rank sum test, *p* = 2.24e−06). Pairwise Dunn’s test showed the genome sizes were significantly different between ‘0.1 µm filter’ and ‘0.2 µm filter’ (fdr adjusted *p* = 1.02e−06), and between ‘0.2 µm filter’ and ‘None’ (fdr adjusted *p* = 9.08e−05). **B** The scatter plot shows the distribution of log_2_ filter enrichment factors (the ratio of average normalized genome coverage in 0.2 µm filter fractions to 0.1 µm filter fractions from metagenomes) of Patescibacteria MAGs, as the function of their genome sizes. The dashed lines indicate the cut-off value of 5 and 1/5 for filter enrichment factors on the y-axis

The average genome size of all Patescibacteria MAGs enriched in the 0.1 µm filter fractions (688.7 ± 139.4 kb) was significantly smaller (Dunn’s test, *p* = 1.02e−06) than that of the Patescibacteria MAGs enriched in the 0.2 µm filter fractions (883.1 ± 204.3 kb), (Fig. 4A). There was no significant difference in the genome completeness and contamination values between the two groups. The filter enrichment factors based on average normalized genome coverages for these Patescibacteria MAGs showed significant positive correlation with their genome sizes (Fig. 4B).

When we analyzed the gene compositions of the two sets of Patescibacteria genomes, the genes encoding type-IV pilus assembly proteins (PilC, PilM, PilO) were significantly overrepresented (two-proportions z-test, *p* = 1.4e−04) in Patescibacteria enriched in the 0.1 µm filter fractions (~ 88% of these genomes) as compared to those from the 0.2 µm filter fractions (~ 64% of these genomes). Similarly, genes encoding cell division proteins FtsW and FtsI were present in 93% and 36% of the Patescibacteria MAGs enriched in 0.1 µm filter fractions, respectively. In comparison, the same genes were present in only 70% and 3% MAGs enriched in the 0.2 µm filter fractions (two-proportions z-test, *p* = 6.2e−04 and 4.7e−04). The gene encoding for the rod-shape determining protein (MreB) was also more likely to be found in Patescibacteria MAGs enriched in the 0.1 µm filter fraction (95% in the 0.1 µm-enriched vs 75% in the 0.2 µm-enriched, two-proportions z-test, *p* = 1.8e−03). Additionally, genes involved in colanic acid biosynthesis (*wcaH* and *wcaF*) were uniquely present in ~ 10% of the Patescibacteria enriched in the 0.1 µm filter fractions.

Conversely, the L-lactate dehydrogenase gene was detected in 12% of the MAGs enriched in the 0.2 µm filter fractions and was entirely absent in the 0.1 µm-enriched MAGs. A similar pattern was found for the tryptophan synthase genes, *trpA* and *trpB*, which were detected in 15% and 18% of the MAGs enriched in the 0.2 µm filter fractions, but absent in Patescibacteria MAGs enriched in the 0.1 µm filter fractions.

### Growth dynamics of Patescibacteria using in situ measure of replication

**Fig. 5:**
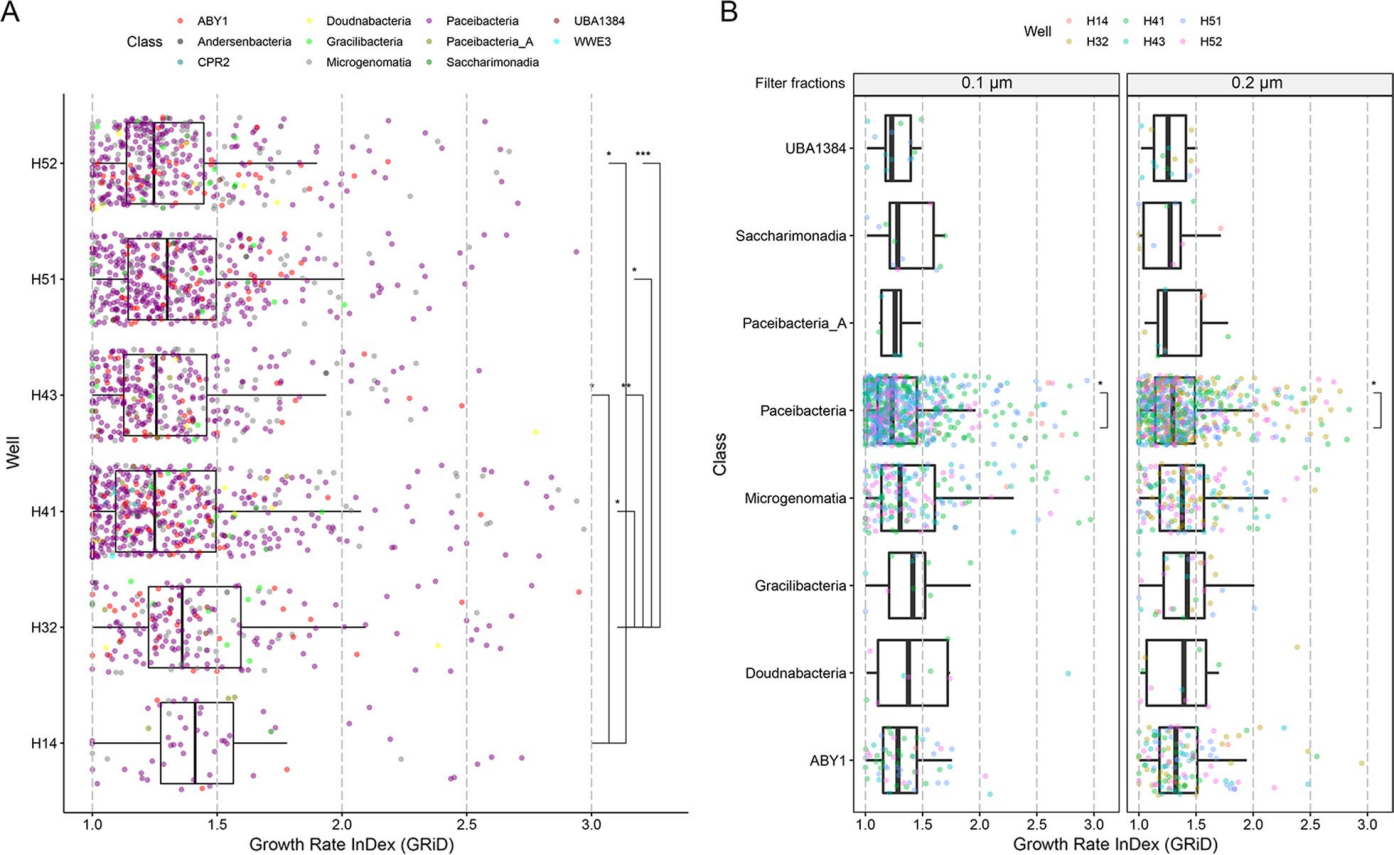
The estimated growth rate index (GRiD) distribution of Patescibacteria MAGs across the metagenomes.** A** Well-wise GRiD distribution of all Patescibacteria. **B** GRiD distribution of classes of Patescibacteria in 0.1 µm and 0.2 µm filter fractions. The statistical significance was calculated by using the t_test function with FDR correction in R package *rstatix* [85]

Patescibacteria MAGs had comparatively higher estimated growth measures (GRiD values) in the near surface wells of the groundwater transect (wells H14 and H32), in comparison to the downstream wells (Fig. 5A). Specifically, these Patescibacteria showed significantly higher GRiD values at well H14 as compared to the downstream wells H41 and H43, and significantly higher GRiD values at well H32 as compared to all other wells present downstream. Notably, the wells with highest mean GRiD values for Patescibacteria were also the wells with lowest number of Patescibacteria MAGs. (Additional file 4: Fig. S1).

The GRiD values were significantly higher (Welch Two Sample t-test, *p* = 8.73e−07) in Patescibacteria MAGs enriched in 0.2 µm filter fractions (1.40 ± 0.27) as compared to Patescibacteria MAGs enriched in the 0.1 µm filter fractions (1.25 ± 0.029). When we compared the GRiD values of individual classes of Patescibacteria between 0.1 and 0.2 µm filter fractions, only MAGs from class Paceibacteria showed significantly higher GRiD values in the 0.2 µm filter fractions (Welch Two Sample t-test, *p* = 6.14e−03, Fig. 5B).

### Limited metabolic and biosynthetic capabilities in Patescibacteria

**Fig. 6:**
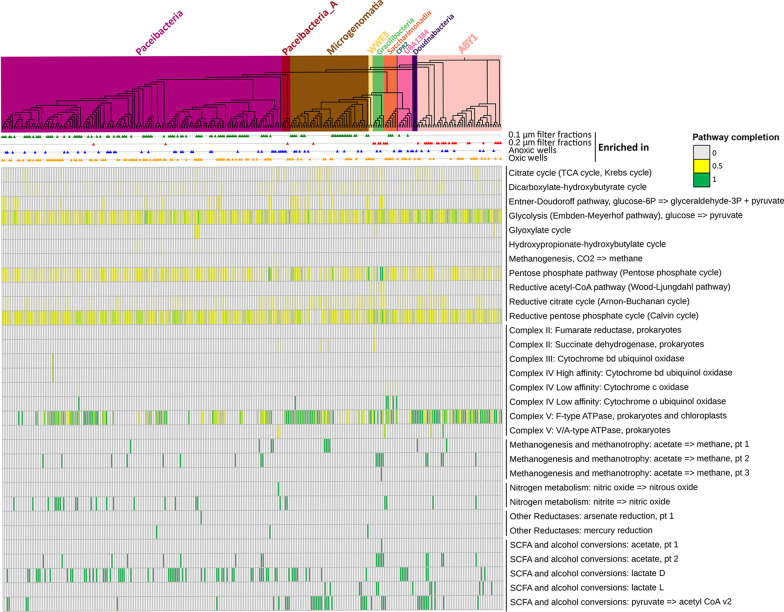
Metabolic and functional repertoire of the high quality Patescibacteria MAGs. The heatmap shows completeness of pathways and presence/absence of the functions in 291 high-quality Patescibacteria genomes annotated within DRAM [72], arranged according to their phylogenetic placement. Clade background colors within the phylogenetic tree represent respective taxonomic classes of Patescibacteria. Colored triangles next to each genome represent their enrichment in 0.1 µm filter fractions (green), 0.2 µm filter fractions (red), anoxic wells (blue) and oxic wells (orange), respectively. Electron transport chain complexes I–IV, sulfur metabolism functions, and photosynthesis related genes were absent from almost all the MAGs. A similar heatmap arranged as per the fivefold enrichment of the MAGs in oxic and anoxic wells is provided as Additional file 5: Fig. S2

Metabolic reconstructions based on KEGG modules revealed that the metabolic repertoire of the analyzed Patescibacteria genomes did not show a clear separation by their taxonomy (Fig. 6) nor followed a particular pattern in oxic and anoxic wells (Additional file 5: Fig. S2). All Patescibacteria MAGs lacked central energy metabolism and biosynthetic pathways for most amino acids and vitamins. The tri-carboxylic acid (TCA) cycle was missing in 81.8% of the Patescibacteria MAGs and was incomplete for the remaining 18.2% of the MAGs. Glycolysis was incomplete in all MAGs, pentose phosphate pathway (PPP) was incomplete in 92% of the MAGs, and reductive PPP was absent in 97% of the MAGs. Biosynthesis pathways for most of the amino acids (except serine, glycine and sometimes asparagine) and vitamins (except cobalamin and thiamin) were missing in most of the Patescibacteria MAGs. In addition, electron transport chain complexes (I–IV) were not identified, with exception of gene encoding for the F-Type ATPase (from ETC complex V) in 59.7% of the Patescibacteria.

However, Patescibacteria possessed some notable genes, namely those coding for copper transporter (*copA*) and cobalt transporter (*corA*) that are usually found in pathogenic bacteria [42, 43]. Also, carbohydrate active enzymes (CAZy) responsible for degradation of starch (11% MAGs), polyphenolics (25% MAGs) and chitin (11% MAGs) were observed. At least 13% of the MAGs had more than one type of CAZy. Patescibacteria also encoded genes for small chain fatty acids (SCFA) and alcohol conversion functions e.g. D-lactate dehydrogenase (25% MAGs), L-lactate dehydrogenase (4% MAGs), and conversion of pyruvate to Acetyl-CoA (K00174, 14% MAGs). Acetate kinase was found in only 6% of the Patescibacteria MAGs. A mutually exclusive presence of D- and L-lactate dehydrogenases was observed.

### Genomic signs of adaptive response of Patescibacteria to oxic and anoxic conditions

We classified 134 Patescibacteria MAGs as fivefold enriched in oxic wells (H32, H41 and H51) and 64 Patescibacteria MAGs as fivefold enriched in anoxic wells (H14, H43 and H52). No taxonomic preference for oxic or anoxic conditions was observed. Patescibacteria MAGs enriched in oxic sites showed some unique features with respect to their ability to resist oxidative stress. We found that superoxide dismutase genes (SOD2, K04564, Fe–Mn family) were encoded by significantly higher proportion (82.8%) of the Patescibacteria MAGs enriched in oxic wells than in anoxic wells (65.6%) (two-proportions z-test, adjusted *p* = 8.8e−03), but there was no evidence for other stress regulator genes (*oxyR*, *soxR*, *soxS*, *rpoS*). There were no relevant metabolic pathways or genes specific to the 64 Patescibacteria MAGs enriched in anoxic wells (Additional file 5: Fig. S2).

Correlation of the genomic coverages (relative abundances) of the Patescibacteria MAGs enriched in oxic wells with the dissolved oxygen concentrations revealed highly significant positive correlations for 28 MAGs (Additional file 6). Most of these MAGs belonged to class *Cand*. Paceibacteria (family UBA1539/*Yonathbacteraceae*) and genus GWC2-37-13 from order UBA1406/*Roizmanbacterales*. Most of these MAGs (82%) carried superoxide dismutase gene (K04564) essential for protection against free superoxide radicals in oxic environments.

**Fig. 7:**
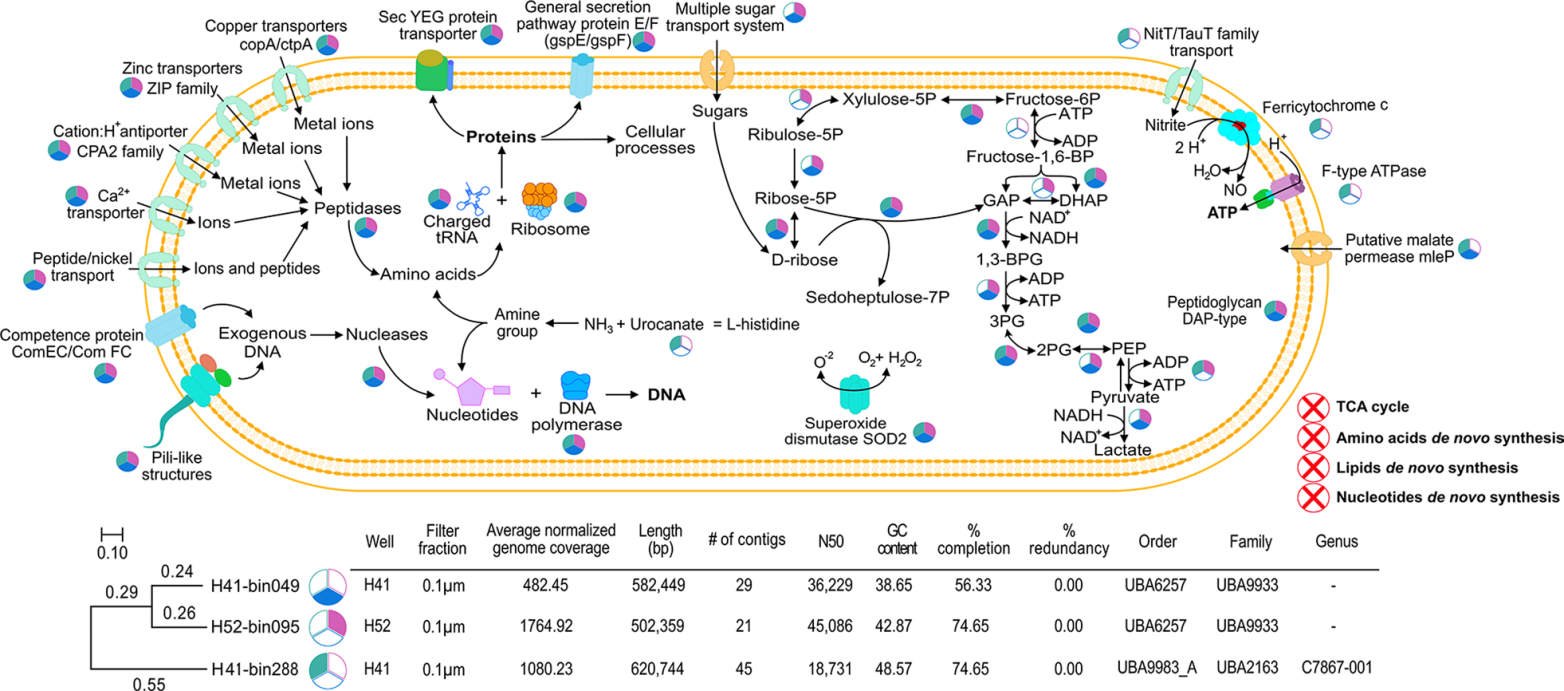
Cell schematic representing the functional repertoire of most abundant model Patescibacteria from oxic and anoxic groundwater wells. The common and genome specific gene features are shown for the three representative genomes based on KEGG pathways. The pie diagrams next to each reaction or function state the presence of respective enzymes or proteins in the three model organisms as per the color key (oxic representatives in green and blue, and the anoxic representative in pink), while absence is indicated by white color

We chose the most abundant, high quality Patescibacteria MAGs from oxic well H41 (H41-bin288, 0.1 µm filter fraction, relative abundance = 0.75% ± 0.15) and anoxic well H52 (H52-bin095, 0.1 µm filter fraction, relative abundance = 2.28% ± 0.37) as model organisms to illustrate the commonalities and divergences in their genomes (Fig. 7). We also included the second most abundant Patescibacteria MAG from the same oxic well H41 (H41-bin049, 0.1 µm filter fraction, relative abundance = 0.41% ± 0.02) from the same taxonomic family as the anoxic representative. This was done to rule out the genomic differences due to the relatively distant evolutionary history of the first pair (H41-bin288 and H52-bin095). The representative MAGs H41-bin288 and H41-bin049 from the oxic well H41 showed positive correlations with oxygen (R = 0.88, *p* = 2.0e−02 and R = 0.75, n.s., respectively), while the representative MAG from anoxic well (H52-bin095) showed a negative correlation (R = − 0.43, n.s.).

Features specific to both representative genomes from oxic well H41 were genes coding for F-type H^+^-transporting ATPase (subunit a, b, c, α, β and γ), NitT/TauT family transporter (involved in transport of inorganic ions like nitrate, sulfonate, and bicarbonate), and nitrite reductase (*nirK* involved in conversion of nitrite to nitric oxide). On the other hand, genes related to sugar sensing and multiple sugar transport systems (ABC.MS.S), and lactate dehydrogenase (fermentation) were specific to the anoxic representative. Common genes or functions were found for all three representative genomes, e.g. genes encoding type IV pilus assembly proteins (PilB, PilC, PilM, and PilO) as well as competence proteins (ComEC, ComFC), useful for DNA uptake from exogenous sources, superoxide dismutase (SOD2) for protection against superoxide radicals, transporters of metal ions like zinc, copper, calcium, nickel. We also identified genes encoding for rod-shape determining proteins, like RodA with additionally related genes encoding for proteins like MreB and MreC in the anoxic representative.

### Co-occurrence patterns of Patescibacteria with other microbial species

**Fig. 8:**
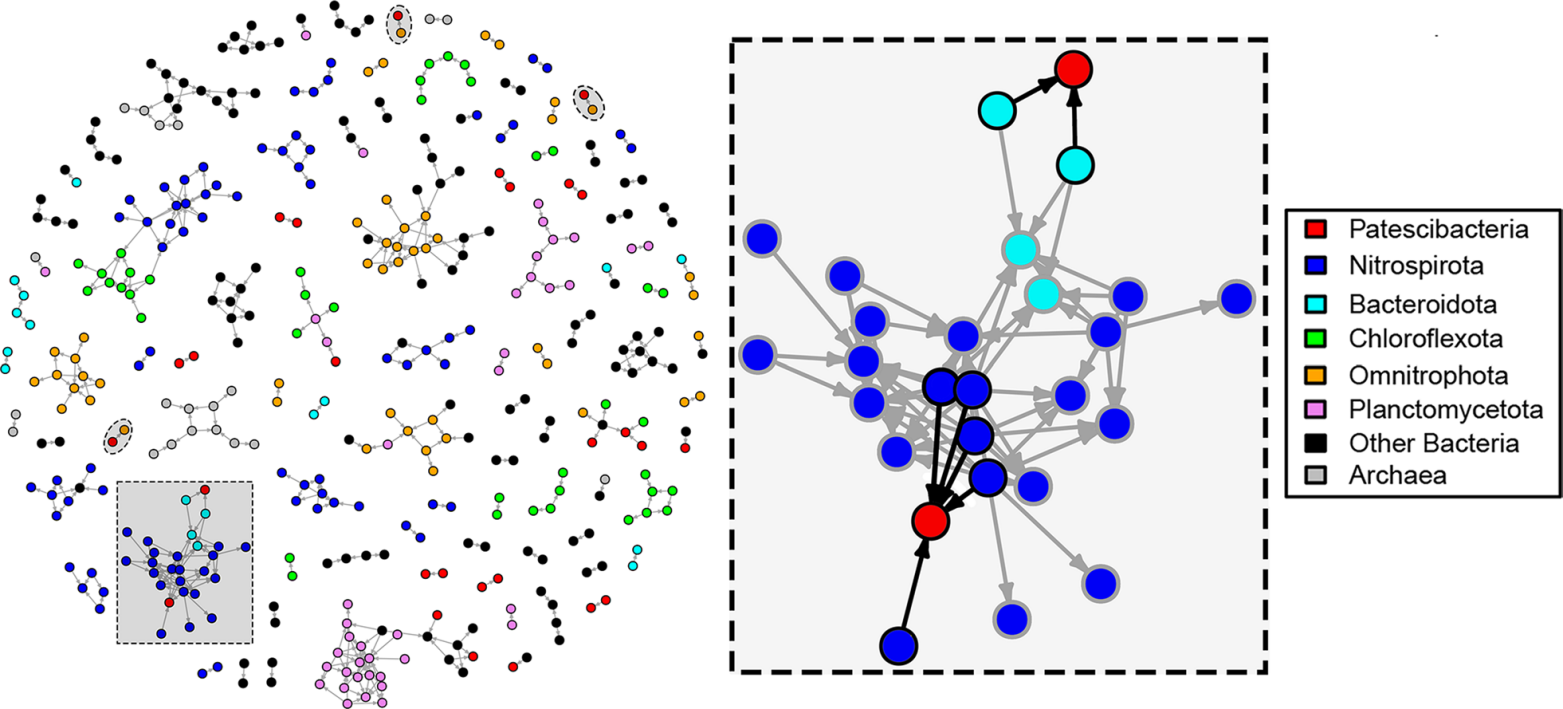
Co-occurrence network among the MAGs recovered from the studied groundwater wells. The proportionality network was constructed using normalized average coverages of the MAGs enriched (by fivefold coverage difference) in 0.2 µm filter fractions as compared to 0.1 µm filter fractions to retain Patescibacteria possibly attached to other microbial hosts. The filled oval regions highlight the direct one-to-one associations of Patescibacteria MAGs paired with Omnitrophota MAGs. The zoomed-in cluster shows direct associations of Patescibcteria MAGs (filled red circles) with multiple Nitrospirota (filled blue circles) and Bacteroidota (filled cyan circles) MAGs highlighted with black outlines and arrows, while grey outlines and arrows indicate indirect associations. For construction of the proportionality network, ⍴ (rho) value cut-off of 0.95 was used

A co-occurrence network generated using metagenomic abundances of MAGs revealed that many species of Patescibacteria were consistently co-occurring with one another, as well as with species of other bacteria and archaea (Fig. 8). The average normalized genome coverages for all the studied MAGs across both filter fractions of all the wells are provided in Additional file 7. The most common one-to-one associations were observed with MAGs from the phyla Nanoarchaeota (mostly order Pacearchaeales), Bacteroidota, MBNT15, and Bdellovibrionota. A small isolated cluster within the network showed indirect but close associations of Patescibacteria with multiple members of the phylum Nitrospirota (genus RGB.16.64.22), and phylum Omnitrophota (Fig. 8).

Under the assumption that Patescibacteria were physically associated with larger host cells, we simplified our co-occurrence network to further refine the associations in the 0.2 µm filter fractions (using the fivefold coverage cut-off as compared to 0.1 µm filter fractions). This follow-up co-occurrence network showed one-to-one associations of MAGs of the phylum Omnitrophota (class koll11) with MAGs from Patescibacteria (each one from the classes Paceibacteria, Microgenomatia, and candidate division ABY1). One of the MAGs from class Paceibacteria showed association with a Proteobacteria MAG (order Rickettsiales), while a MAG from candidate division ABY1 showed direct connections with two Bacteroidota MAGs. Another MAG from class Gracilibacteria showed direct connections with 5 Nitrospirota MAGs from the same genus UBA1546 (Fig. 8). The sequence coverages of these highlighted genome pairs or clusters across the metagenomes are compared in Additional file 8: Fig. S3 and Additional file 9: Fig. S4. Two Actinobacteria MAGs belonging to the species *Aurantimicrobium* sp003194085 also showed associations with Patescibacteria. The first *Aurantimicrobium* co-ocurred with a Patescibacteria (*Cand*. Paceibacteria) MAG, and the second with multiple Patescibacteria (2 *Cand*. Paceibacteria, 2 *Cand*. Gracilibacteria and 3 candidate division ABY1) MAGs.

When we searched for sequence similarity of all gene open reading frames (ORFs) from all Patescibacteria MAGs to ORFs from all other bacterial and archaeal MAGs in the present study using blastn [44], we found various ORFs from other taxa highly similar to Patescibacteria ORFs (95% sequence identity covering 85% length of the query and hit sequences). The most ORFs that matched were between members of genus UBA10092 of Patescibacteria (class Paceibacteria) and two members of the family UBA12090 of Omnitrophota (34 and 16 ORFs, respectively). They included genes encoding for twitching motility protein PilT (K02669), P-type Cu + transporter (K17686) and lipopolysaccharide export system permease protein (K11720). Between members of genus UBA11707 of Patescibacteria (class ABY1) and genus UBA1573 of Proteobacteria (family Micavibrionaceae), 14 such ORFs, including gene encoding for ABC-2 type transport system ATP-binding protein (K01990), were observed. Thirteen such ORFs, including gene for ABC-2 type transport system permease protein (K01992), were observed between members of the family Zambryskibacteraceae of class Paceibacteria and genus ASMP01 of Nanoarchaeota.

To have an idea about the temporal co-occurrence patterns of other groundwater microbes with Patescibacteria, we additionally utilized time-series data based on 16S rRNA gene amplicon sequencing from the same groundwater transect from three wells (H41, H43 and H52) measured over more than six years [45]. We observed that Patescibacteria co-occurred mostly with members of phyla Proteobacteria (mostly order Burkholderiales) and Nitrospirota (order Thermodesulfovibrionia), in the well H41; Verrucomicrobiota, in the well H43 and Planctomycetota (mostly genus *Brocadia*) in the well H52. Some of these reported co-occurrences could also be observed in our MAG based co-occurrence network where a Patescibacteria MAG was identified to co-occur with multiple Nitrospirota (order Thermodesulfovibrionia) MAGs.

## Discussion

Our comprehensive metagenomic analyses revealed that modern pristine groundwater of the Hainich CZE is clearly dominated by *Cand*. Patescibacteria with an average relative abundance of 50% across all wells and a maximum of 79% in the 0.1 µm filter fraction. Compared to other groundwater communities dominated by CPR bacteria ranging from 2–28% [16], 3–40% [18], 10–28% [7] and 36–65% [15], the exceptionally high abundance of CPR bacteria discovered in this study is distributed over distinct geochemical zones spanning oxic and anoxic conditions [17, 37]. Although the spatial distribution patterns of the different *Cand*. Patescibacteria taxa (Fig. 2) were less pronounced than those observed in other bacteria in groundwater of the Hainich CZE [37, 45], and despite their streamlined genomes, we could highlight certain environmental preferences of the *Cand*. Patescibacteria. Access to 587 manually curated MAGs of *Cand*. Patescibacteria, assigned to different filter fractions, allowed us to shed some light on genomic characteristics linked to their cell size and a putative free living or host attached lifestyle.

Patescibacteria have been described mostly in anoxic or hypoxic environments [46, 47]. Our data show no major metabolic or taxonomic differences in Patescibacteria enriched in oxic and anoxic groundwater wells. Significantly higher proportion of superoxide dismutase genes in Patescibacteria MAGs enriched in oxic groundwater wells compared to those in anoxic wells is an example of spatial differentiation that might be due to an environmental selection mechanism, as these enriched species have an advantage to withstand the presence of oxygen radicals when exposed to high O_2_ concentrations. More than 80% of the Patescibacteria MAGs enriched in oxic wells could potentially resist superoxide radicals, and more than 20% showed a positive correlation to oxygen concentrations, in particular those belonging to class *Cand*. Paceibacteria (family UBA1539/*Yonathbacteraceae*) and to order UBA1406/*Roizmanbacterales*. But even closely related Patescibacteria species showed different preferences for oxygen concentrations in terms of metabolic pathways (Fig. 7).

The permanently high O_2_ concentration in well H32 (2.23 ± 0.56 mg/L) and especially in well H41 (4.83 ± 1.7 mg/L) [45, 48], did not lead to enrichment of groundwater Patescibacteria MAGs with genetic traits of energy harvesting mechanisms through aerobic respiration. Exposure to oxygen is not exceptional for *Cand.* Patescibacteria, as oxic soils are the main source for their vertical translocation into shallow groundwater [17, 24]. *Cand.* Patescibacteria represent only 0.55% of the total bacterial soil community in the preferential forest surface-recharge area of the Hainich CZE [17]. Despite this low abundance, these ultra-small organisms are readily mobilized from soil, especially during winter months when ionic strength of the seepage is very low (Herrmann et al. 2021, unpublished observations), and as such constitute the largest fraction of taxa shared between seepage and shallow groundwater [17].

The most abundant Patescibacteria MAG from oxic well H41 (H41-bin288) had genes that encode for nitrite transport and its subsequent reduction into nitric oxide involving ferricytochrome c. Also, this genome possessed a gene for F-Type ATPase to generate energy by ATP formation and it did not encode genes for fermentation (L- or D-lactate dehydrogenase). This collectively suggests the possibility of an alternative anaerobic respiration mechanism in this particular genome. Despite the low in situ concentrations of nitrite, it might be alternatively provided by the nitrification process. This relates to the fact that well H41 is characterized as a nitrification hotspot with measured rates of 0.48 ± 0.09 and 0.64 ± 0.39 nmol NO_*x*_ L^−1^ h^−1^ [49] and to the high relative abundances of *Nitrospira* on the metagenome level and *Thaumarchaeota* on the metatranscriptome level [50]. Presence of genes coding for multiple subunits of F-Type (H^+^ transporting) ATPase in this genome confirms the existence of supplementary ATP synthesis machinery, which are commonly observed in aerobic bacteria [51]. Similarly, notable features specific to both representative genomes from oxic well H41 included genes involved in the transport of inorganic ions like nitrate, sulfonate, and bicarbonate.

The almost complete absence of the aerobic respiration machinery i.e. the electron transport chain complexes, terminal oxidases/electron acceptors, and gene products associated with the TCA cycle, along with widespread presence of L- or D-lactate dehydrogenases confirms the previously postulated fermentative lifestyles of Patescibacteria [11, 15, 52] in members of the three lineages OD1 (Parcubacteria), OP11 (Microgenomates), and BD1-5 (Gracilibacteria). Parcubacteria were proposed to produce acetate, ethanol, lactate, and hydrogen as fermentation products based on metagenomic and proteomic analysis [3, 15, 52]. Presence of L- or D-lactate dehydrogenase genes in one third of the Patescibacteria MAGs indicates specificity for fermentation substrates. In one tenth of the MAGs enriched in 0.1 µm filter fractions, specificity for L-lactate could be observed based on the exclusive presence of L-lactate dehydrogenase genes. Presence of multiple carbohydrate active enzymes (CAZy) in many Patescibacteria suggests their potential for degradation of multiple complex compounds like starch, chitin, and polyphenolics.

The spatial differentiation of *Cand.* Patescibacteria could also be indirectly caused by the preference of a putative host organism for certain environmental conditions. The oxic, nitrate-rich (15.71 mg/L) groundwater of well H41 was dominated by Nitrospirota MAGs, and 5 of them co-occurred with a single Patescibacteria MAG (H52-bin081_1, *Cand.* Gracilibacteria) and had similar abundance patterns (Additional file 9: Fig. S4). As some Nitrospirota MAGs (n = 51) were enriched exclusively in oxic wells, their preference might have determined the distribution pattern of putative CPR episymbionts. Nitrospirota species were also found to be consistently co-occurring with Patescibacteria in some of the studied wells based on OTU abundances from 16S rRNA gene amplicon sequencing data collected over 6.5 years [45] as well as MAG abundances from this study across the groundwater transect. At the minimum, these observations suggest common niche preferences between some members of these two phyla.

To elucidate other possible associations of Patescibacteria with other prokaryotes, we utilized above mentioned time-series data that revealed consistent co-occurrence of Patescibacteria OTUs with OTUs from Proteobacteria, Verrucomicrobiota, and Planctomycetota in addition to OTUs from Nitrospirota [45]. There are in silico predictions of acquisition of few unique genes by human-associated CPR bacteria (Saccharibacteria) during mammalian host adaptation [53]. Although there is no strong evidence of lateral gene transfer events even in experimentally confirmed Patescibacteria-host pairs, we searched within the genomic characteristics of all Patescibacteria and all other MAGs, we found various ORFs from other taxa highly similar with Patescibacteria, between members of (1) class Paceibacteria and family Omnitrophota, (2) class ABY1 and family Micavibrionaceae, and (3) family Zambryskibacteraceae of class Paceibacteria and genus ASMP01 of Nanoarchaeota, suggesting probable acquisition of motility and transport functions from other bacteria or archaea.

Network analysis based on abundances of all MAGs of both filter fractions revealed that the members of the phyla Bacteroidota, MBNT15, and Bdellovibrionota along with members of phyla Nitrospirota and Omnitrophota had direct specific connections with some Patescibacteria. Furthermore, we restricted the network analysis only to MAGs enriched on the 0.2 µm filter fractions (57 Patescibacteria and 423 other MAGs) in order to identify Patescibacteria that would be potentially attached to other larger host cells. This narrowed-down analysis showed interactions of Patescibacteria with few specific MAGs of the phyla Bacteroidota, Nitrospirota, Omnitrophota, and Actinobacteria. Our co-occurrence analysis did not reveal direct connections of Actinobacteria MAGs with any of the Saccharibacteria, although Actinobacteria are reported as host for Saccharibacteria (TM7) in human oral cavity and wastewater foam [12, 29, 31, 54]. However, direct network connections of *Aurantimicrobium* species, members of the phylum Actinobacteria with multiple other Patescibacteria MAGs from classes Paceibacteria, Gracilibacteria, and candidate division ABY1 hint towards possible host-symbiont relationships in these particular pairs.

Direct one-to-one connections with members of other phyla were found in only 5 out of 57 (8.77%) Patescibacteria MAGs enriched in 0.2 µm filter fractions, suggesting that the majority of groundwater Patescibacteria of the Hainich CZE is not specifically associated with one single host, but associations with multiple hosts cannot be ruled out. The attachments between cells are often fragile and may be partly or completely disrupted during filtration and sample processing steps, and hence are difficult to track using sequential filtration. An even lower percentage of associations (< 1.5%) based on potentially co-sorted SAGs containing DNA from heterogeneous sources was reported from Beam et al. [11].

We also detected a few Eukaryotes in our metagenomes based on a high proportion of reads mapped to the 18S rRNA database, including orders Euglenozoa, Opisthokonta, Ciliophora. Some higher organisms might also serve as possible hosts to some Patescibacteria as reported in other environments [28]. However, no high quality eukaryotic genomes could be resolved from the given metagenome assemblies, and hence could not be included in co-occurrence analysis.

On average, Patescibacteria enriched in 0.1 µm filter fractions had 22% smaller genome size than those enriched in 0.2 µm filter fractions, and it has been previously shown that smaller cell size is linked to genome reduction [55, 56]. This genome size difference might be due to differences in average cell sizes of *Cand*. Paceibacteria and *Cand*. Microgenomatia that were preferentially enriched within 0.1 µm filter fractions; and candidate division ABY1, and *Cand*. Gracilibacteria that were preferentially enriched within the 0.2 µm filter fractions. Smaller genomes in tiny CPRs might be the result of genome streamlining leading to lack of complex energy metabolism and biosynthetic capabilities which makes them rely on other cells through cell–cell attachment.

We found Type IV pilus assembly proteins in a higher proportion of Patescibacteria enriched in 0.1 µm filter fractions. These proteins are responsible for formation of pilin-like appendages that are involved in a variety of functions like adherence to host cells, locomotion, DNA uptake as well as protein secretion in bacteria [57], which would support physical association with other microbes. Type IV pili (T4P) are essential for virulence of some Gram-negative pathogenic bacteria [58] and also found in Gram-positive bacteria with a different pilus assembly mechanism involving a sortase [59]. Pili like appendages were microscopically shown to form surface attachment of CPR bacteria with other (host) large cells [18]. The symbiotic association of TM7i (*Cand*. Saccharibacteria) with its host *Leucobacter aridocollis* J1, mediated by T4P was identified in a co-culture experiment [60]. As pilus mediated attachments are often fragile, small Patescibacteria cells passing through the 0.2 µm filters do not necessarily indicate lack of cell–cell attachment with larger bacterial cells. Many of these ultra-small Patescibacteria appear to have a rod-shaped morphology, as genes encoding the rod shape-determining protein (MreB) were found in a higher proportion of MAGs enriched in 0.1 µm filter fractions. The recent reconstruction of the last bacterial common ancestor (LBCA) genome of CPR lineage suggests a rod-shaped morphology [61]. However, most of the reported morphologies for the Patescibacteria are cocci [12, 18, 21]. Although we cannot rule out that some of the larger rod-shaped Patescibacteria could still pass through the 0.2 µm filter pores, this would not explain the enrichment in the 0.1 µm filter fractions. More direct microscopic visualization is needed to verify the morphology of these ultra-small Patescibacteria.

We found higher growth rates of Patescibacteria in near-surface wells (H14, H32) of the groundwater transect than in the ones more downstream. Growth of CPR bacteria is stimulated after attachment to host-cells [18]. As cell–cell aggregations might be more prone to dispersal limitations in a dense rock matrix, surface-near wells could have higher probabilities of host interactions. But our co-occurrence analysis did not reveal direct connections of CPR MAGs with higher growth rates with other MAGs.

Groundwater of the very shallow well H14, located uphill of the transect, shows a fast response to weather events [62], and is characterized by both the highest bacterial diversity and the presence of well-known surface heterotrophs; whereas core groundwater species dominated groundwater microbiomes in the downstream direction [37]. This well, along with the other near-surface well (H32) showed the lowest relative abundances of Patescibacteria and of Patescibacteria MAGs, although those that were detected had higher expected replication rates on average. A possible explanation for this pattern is that surface exported members were replicating within the soil before being flushed into the groundwater. Other, more successful groundwater CPR groups may have slower growth and replication rates within the transect due to much lower microbial cell densities and less available organic carbon. Indeed, some taxa such as those belonging to *Cand.* Saccharimonadia, which had among the highest growth rates, did not flourish within other wells of the groundwater transect. We hypothesize that they might be more adapted to soil habitats compared to groundwater, which was also observed in previous studies [17].

The predominance of particular CPR species in oxic (H41) and anoxic (H52) wells appears to be the result of environmental preference or exploitation of other organisms for cellular requirements in the nutrient deficient groundwater. Some potential hosts supporting an episymbiotic lifestyle could be identified. The environmental preference of some of these hosts, e.g. Nitrospirota for oxygen and nitrogen in well H41, would explain the predominance of their potential Patescibacteria episymbiont in H41, with an estimated episymbiont-to-host ratio of 3.6:1 based on coverages of Patescibacteria and Nitrospirota MAGs in total coverage of all binned genomes. But the vast majority of the ultra-small Patescibacteria in the groundwater appears to be free-living, self-sufficient with their minimal genomes [11, 46], adapted to oligotrophic conditions with low growth rates, and equipped with genes to cope with oxidative stress only if needed. We found evidence that the majority has the capability to attach to other cells, which appears to also include other Patescibacteria, and this attachment might be not very specific or for longer time periods, just long enough to loot or exchange supplies.

## Conclusions

The Candidate Phyla Radiation represent the largest phylogenetic diversity within the bacterial domain, which has not been reflected in the metabolic versatility of genomic representatives studied to date. Here we leveraged a well characterized aquifer transect, that is dominated by members of the CPR and spans large biogeochemical gradients, to explicitly explore genomic adaptations to environmental conditions. The most significant and surprising result was the high level of similarity in predicted metabolic functions and expected lifestyles that spanned large redox gradients from fully oxic to completely anoxic groundwater, both within the larger CPR clade as well as at finer phylogenetic resolutions. One noteworthy exception was a differential abundance in superoxide dismutase, a potentially useful indicator of oxygen exposure in CPR genomes recovered from other environments or already deposited to sequence databases. Due to a suspected dependence on other bacterial hosts, we searched among > 1200 constructed MAGs and a larger amplicon dataset for potential partners, finding that only 8% of CPR MAGs exhibited significant one-to-one relationships. Therefore, we speculate that most members of the CPR might form non-specific attachments to multiple hosts to supplement their energetic demands within oligotrophic groundwaters.

## Materials and methods

### Groundwater sampling, DNA extraction and sequencing

Samples were collected from a groundwater transect system spanning through a ~ 6 km long zone including forest, pasture and agricultural land within the Hainich Critical Zone Exploratory (CZE) located in Thuringia, Germany. The Hainich CZE, established and extensively studied by Collaborative Research Center AquaDiva [36], accesses a hillslope groundwater flow system of thin-bedded marine sediments (Muschelkalk, German Triassic). The lower aquifer (HTL) is characterized by a considerable degree of karstification in its bioclastic limestone beds (Trochitenkalk Fm.) that favor fast groundwater flow, whereas the hanging upper aquifers (HTU) of the Meissner Fm., Warburg Fm., and Keuper deposits feature a slower water flow [48]. The groundwater was collected from 6 wells (H14, H41, H43, H51, H52 in January 2019 and H32 in November 2018) spanning various zones of the transect. For each well, on average 61.3 ± 35.4 L of groundwater was filtered through 0.2 µm filters (Omnipore Hydrophilic PTFE membrane, Merck Chemicals GmbH) followed by 0.1 µm filters in triplicates (except for well H32 where there were only two replicates out of which one from the November 2018 sampling campaign was used as biological replicate). All the 32 filter fractions were immediately frozen and stored under − 80 °C. The DNA was extracted from each filter using a phenol/chloroform protocol, the libraries generated with an NEBNext Ultra FS DNA preparation kit, and sequenced on an Illumina NextSeq 500 system with paired-end library (2 × 150 bp).

On an average 9.8 ± 1.15 Gb of raw DNA sequence data were obtained from each of the 32 filter fractions. Of which, 86.12 ± 0.57% of the reads were of very high quality (at least quality score Q40). Subsequent quality control steps like adapter trimming, PhiX detection and removal using BBDuk (bbtools version 37.09, written by Brian Bushnell, last modified March 30, 2017) further improved the quality of the reads. These high-quality reads were then used for metagenomic assembly and followed by genome binning steps.

### Metagenomic assembly, genome binning and refinement

The quality controlled reads of each individual filter fraction replicate were assembled and scaffolded using metaSPAdes v3.13 [63]. Scaffolds larger than 1 kb were used for downstream analyses. Genome binning was carried out using three binning algorithms—Abawaca v1.07 [15], ESOM [64, 65] and Maxbin2 v2.2.4 [66]. The values 3000 and 5000 bp as well as 5000 and 10,000 bp were used as *-min* and *-max* parameters to calculate 4-mer frequencies for Abawaca and ESOM (the script esomWrapper.pl, https://github.com/tetramerFreqs/Binning), and both the 40 and 107 marker gene sets were utilized in Maxbin2. DASTool v1.1 [14] was used to determine the best bins among these approaches. Bins were further refined manually inside the Anvi’o workflow v6.1 [39, 40]. The quality of the refined genome bins (completeness and contamination/redundancy) was calculated based on domain-level bacterial/ archaeal single-copy core genes within Anvi’o. To estimate the completeness and contamination of CPR genomes, we used 43 CPR specific markers from Brown et al., 2015 [15] within CheckM [67]. Genomes from each assembly were de-replicated using dRep v2.6.2 [68] at 99% ANI to remove strain level redundancy across sites, resulting into 1275 representative MAGs. Genome coverages were calculated within Anvi’o, and were normalized using number of RNA polymerase B (*rpoB*) genes identified within the metagenomic reads.

### Taxonomic assignments, gene annotations and pathway predictions

Overall community composition of each metagenome was determined using phyloFlash v3.4 [69] based on proportions of reads mapped to SILVA SSU rRNA Ref NR99 database, Release 138 [38]. Taxonomic classification of individual MAGs was performed by GTDB-Tk v0.3.2 [70] using GTDB Release 89 as reference database. Out of the 1275 genomes GTDB-Tk classified 587 genomes as *Cand*. Patescibacteria at phylum level. We used *anvi-script-gen-CPR-classifier* script from Anvi’o v6.1 [39, 40] which uses supervised machine learning model (random forest classifier) to train the program and *anvi-script-predict-CPR-genomes* for predicting the probability of the MAGs to confirm the CPR genomes. The training is based on the profile of previously published 139 single copy core genes from hundreds of CPR genomes from Brown et al*.* [15] and Campbell et al*.* [41] as input. This model confirmed 291 out of 587 genomes as CPR with a high confidence score (75% or more). While the model was inconclusive in case of the remaining 296 genomes based on low confidence score (less than 75%).

The gene annotations, coding sequences, respective protein sequences, coverage calculations and other mapping statistics for all the genomes were exported by *anvi-summerize* program from within the Anvi’o workflow. The annotations were also carried out using Prodigal v2.6.3 [71]. Distilled and Refined Annotation of Metabolism (DRAM) [72] was used to generate pathway/metabolism summaries. At least one proper (other than hypothetical, uncharacterized or gene with unknown function) annotation from KEGG [73], MEROPs [74], Pfam [75] or dbCAN [76] was considered. This generated a single tab delimited annotation file listing the best hits from all these databases as well as summaries focused on most important pathways and functions. The pathway coverages (completeness) of central metabolism pathways were calculated based on KEGG modules definitions (https://www.genome.jp/kegg/module.html).

### Phylogenetic analysis

Single copy core bacterial genes were detected in all the 1087 bacterial MAGs using hmm profile (default ‘Bacteria_71’ hmm profile in Anvi’o v6.1), their protein sequences were extracted and aligned using MUSCLE [77] from within the Anvi’o [39, 40]. A phylogenetic tree based on multiple sequence alignment of the 68 core proteins present in all bacterial MAGs (1087) was constructed using Approximate Maximum Likelihood in FastTree v2.1.11 SSE3, OpenMP [78] with 1000 bootstrap replications. The subset of the tree was used for arranging the metabolic pathways of 291 selected Patescibacteria MAGs in Fig. 6.

### In-situ measurement of replication

The forward sequencing reads from all the metagenomes were mapped to the MAGs to calculate the sequence coverage of individual contigs. These coverage profiles were utilized to calculate Growth Rate InDex (GRiD) [79] which is directly proportional to the growth rates of the cells in a given environment. GRiD measures the difference in genome copies closer to the origin of replication compared to the terminus caused by ongoing replication forks. The coverage cut-off of 0.7 was used to remove extremely low coverage contigs.

### Statistical analyses

The difference in the mean genome sizes of the MAGs enriched in different filter fractions were compared using Kruskal–Wallis rank sum test followed by pairwise Dunn’s test in R [80]. The proportions of gene annotations (KEGG) in the MAGs enriched in different filter fractions or oxic and anoxic wells were compared with two-proportions z-test with Yates' continuity correction in R. The *p* values were adjusted for multiple testing using ‘fdr’ correction unless otherwise mentioned.

### Co-occurrence network analysis

We used normalized average genome coverages of all the 1275 MAGs across all the metagenomes as the approximation of abundance profiles of species from respective metagenomes. This abundance matrix was used to calculate proportionality of the coverage profiles in R package propR v4.2.6 [81]. A *⍴* cutoff of 0.95 was used for network creation to highlight only the most relevant co-occurrences. The network was generated using the R package igraph v1.2.6 [82] and exported to Cytoscape v3.8.2 [83] for visualization using R package Rcy3 v2.8.1 [84].

### Search for ORF similarity

We carried out blastn [44] search on all the annotated ORFs for Patescibacteria MAGs as a query against all the ORFs of all the MAGs other than Patescibacteria. We filtered the results based on 95% sequence identity over 95% query and hit ORF length with e-value cut off of 1.0e−5. We chose only one hit in case of more than one hits for the same query sequence.

## Supplementary Information

**Additional file 1.** Single copy genes from publically available CPR genomes used to predict CPR MAGs in this study. The file was taken from Anvi’o codebase (https://github.com/merenlab/anvio).

**Additional file 2.** Genome statistics and taxonomic assignments of Patescibacteria MAGs in this study.

**Additional file 3.** The Newick tree file for phylogenetic tree shown in Fig. 3B.

**Additional file 4: Fig. S1.** Correlation of average normalized genome coverages of Patescibacteria MAGs from respective wells with respective GRiD values.

**Additional file 5: Fig. S2.** Metabolic and functional repertoire of high quality Patescibacteria MAGs. The heatmap shows completeness of pathways and presence/absence of functions in 291 high-quality Patescibacteria genomes annotated with DRAM, arranged according to their enrichment in oxic and anoxic wells based on 5-fold coverage criterion.

**Additional file 6.** Correlations of average normalized genome coverages of Patescibacteria MAGs enriched in oxic wells with dissolved oxygen and nitrate concentration.

**Additional file 7.** Genomic coverages of 1275 microbial MAGs in all studied metagenomes.

**Additional file 8: Fig. S3.** Coverage distribution of selected MAGs from the network in Fig. 8. Only the direct one-to-one pairs of Patescibacteria with other MAGs are plotted.

**Additional file 9: Fig. S4.** Coverage distribution of selected MAGs from the highlighted cluster in network in Fig. 8. Only the direct connections of Patescibacteria with other MAGs are plotted.

## Data Availability

Data used for this study were deposited into the European Nucleotide Archive (ENA). The raw metagenomic sequencing reads for the studied samples were deposited under ENA project accession PRJEB36505, and assemblies for individual samples/MAGs were deposited under ENA project accession PRJEB36523. All Patescibacteria/CPR MAGs used for this study are available from Open Science Framework (OSF) repository: https://osf.io/wq7tr/.

## References

[1] Hug LA, Baker BJ, Anantharaman K, Brown CT, Probst AJ, Castelle CJ (2016). A new view of the tree of life. Nat Microbiol.

[2] Elshahed MS, Najar FZ, Aycock M, Qu C, Roe BA, Krumholz LR (2005). Metagenomic analysis of the microbial community at Zodletone Spring (Oklahoma): insights into the genome of a member of the novel candidate division OD1. Applied and environmental microbiology. Am Soc Microbiol.

[3] Wrighton KC, Thomas BC, Sharon I, Miller CS, Castelle CJ, VerBerkmoes NC (2012). Fermentation, hydrogen, and sulfur metabolism in multiple uncultivated bacterial phyla. Science.

[4] Mohiuddin MM, Salama Y, Schellhorn HE, Golding GB (2017). Shotgun metagenomic sequencing reveals freshwater beach sands as reservoir of bacterial pathogens. Water Res.

[5] Rinke C, Schwientek P, Sczyrba A, Ivanova NN, Anderson IJ, Cheng J-F (2013). Insights into the phylogeny and coding potential of microbial dark matter. Nature.

[6] Probst AJ, Castelle CJ, Singh A, Brown CT, Anantharaman K, Sharon I (2017). Genomic resolution of a cold subsurface aquifer community provides metabolic insights for novel microbes adapted to high CO(2) concentrations. Environ Microbiol.

[7] Probst AJ, Ladd B, Jarett JK, Geller-McGrath DE, Sieber CM, Emerson JB (2018). Differential depth distribution of microbial function and putative symbionts through sediment-hosted aquifers in the deep terrestrial subsurface. Nat Microbiol.

[8] Correa-Galeote D, Bedmar EJ, Fernández-González AJ, Fernández-López M, Arone GJ (2016). Bacterial communities in the rhizosphere of amilaceous maize (*Zea mays* L.) as assessed by pyrosequencing. Front Plant Sci.

[9] Frey B, Rime T, Phillips M, Stierli B, Hajdas I, Widmer F (2016). Microbial diversity in European alpine permafrost and active layers. FEMS Microbiol Ecol.

[10] Wurzbacher C, Nilsson RH, Rautio M, Peura S (2017). Poorly known microbial taxa dominate the microbiome of permafrost thaw ponds. ISME J.

[11] Beam JP, Becraft ED, Brown JM, Schulz F, Jarett JK, Bezuidt O (2020). Ancestral absence of electron transport chains in Patescibacteria and DPANN. Front Microbiol.

[12] He X, McLean JS, Edlund A, Yooseph S, Hall AP, Liu S-Y (2015). Cultivation of a human-associated TM7 phylotype reveals a reduced genome and epibiotic parasitic lifestyle. Proc Natl Acad Sci USA.

[13] Baker JL, Morton JT, Dinis M, Alvarez R, Tran NC, Knight R (2021). Deep metagenomics examines the oral microbiome during dental caries, revealing novel taxa and co-occurrences with host molecules. Genome Res.

[14] Shaiber A, Willis AD, Delmont TO, Roux S, Chen L-X, Schmid AC (2020). Functional and genetic markers of niche partitioning among enigmatic members of the human oral microbiome. Genome Biol.

[15] Brown CT, Hug LA, Thomas BC, Sharon I, Castelle CJ, Singh A (2015). Unusual biology across a group comprising more than 15% of domain Bacteria. Nature.

[16] Danczak R, Johnston M, Kenah C, Slattery M, Wrighton KC, Wilkins M (2017). Members of the Candidate Phyla Radiation are functionally differentiated by carbon-and nitrogen-cycling capabilities. Microbiome.

[17] Herrmann M, Wegner C-E, Taubert M, Geesink P, Lehmann K, Yan L (2019). Predominance of Cand. Patescibacteria in groundwater is caused by their preferential mobilization from soils and flourishing under oligotrophic conditions. Front Microbiol.

[18] He C, Keren R, Whittaker ML, Farag IF, Doudna JA, Cate JH (2021). Genome-resolved metagenomics reveals site-specific diversity of episymbiotic CPR bacteria and DPANN archaea in groundwater ecosystems. Nat Microbiol.

[19] Vigneron A, Cruaud P, Langlois V, Lovejoy C, Culley AI, Vincent WF (2020). Ultra-small and abundant: candidate phyla radiation bacteria are potential catalysts of carbon transformation in a thermokarst lake ecosystem. Limnol Oceanogr Lett.

[20] Vavourakis CD, Andrei A-S, Mehrshad M, Ghai R, Sorokin DY, Muyzer G (2018). A metagenomics roadmap to the uncultured genome diversity in hypersaline soda lake sediments. Microbiome.

[21] Luef B, Frischkorn KR, Wrighton KC, Holman H-YN, Birarda G, Thomas BC (2015). Diverse uncultivated ultra-small bacterial cells in groundwater. Nat Commun.

[22] Castelle CJ, Banfield JF (2018). Major new microbial groups expand diversity and alter our understanding of the tree of life. Cell.

[23] Gleeson T, Befus KM, Jasechko S, Luijendijk E, Cardenas MB (2016). The global volume and distribution of modern groundwater. Nat Geosci.

[24] Krüger M, Potthast K, Michalzik B, Tischer A, Küsel K, Deckner FF (2021). Drought and rewetting events enhance nitrate leaching and seepage-mediated translocation of microbes from beech forest soils. Soil Biol Biochem.

[25] Starr EP, Shi S, Blazewicz SJ, Probst AJ, Herman DJ, Firestone MK (2018). Stable isotope informed genome-resolved metagenomics reveals that Saccharibacteria utilize microbially-processed plant-derived carbon. Microbiome.

[26] Nicolas AM, Jaffe AL, Nuccio EE, Taga ME, Firestone MK, Banfield JF (2021). Soil Candidate Phyla Radiation bacteria encode components of aerobic metabolism and co-occur with nanoarchaea in the rare biosphere of rhizosphere grassland communities. Msystems.

[27] Albertsen M, Hugenholtz P, Skarshewski A, Nielsen KL, Tyson GW, Nielsen PH (2013). Genome sequences of rare, uncultured bacteria obtained by differential coverage binning of multiple metagenomes. Nat Biotechnol.

[28] Gong J, Qing Y, Guo X, Warren A (2014). “Candidatus Sonnebornia yantaiensis”, a member of candidate division OD1, as intracellular bacteria of the ciliated protist *Paramecium bursaria* (Ciliophora, Oligohymenophorea). Syst Appl Microbiol.

[29] Bor B, Collins A, Murugkar P, Balasubramanian S, To T, Hendrickson E (2020). Insights obtained by culturing Saccharibacteria with their bacterial hosts. J Dent Res.

[30] Cross KL, Campbell JH, Balachandran M, Campbell AG, Cooper SJ, Griffen A (2019). Targeted isolation and cultivation of uncultivated bacteria by reverse genomics. Nat Biotechnol.

[31] Murugkar PP, Collins AJ, Chen T, Dewhirst FE (2020). Isolation and cultivation of candidate phyla radiation Saccharibacteria (TM7) bacteria in coculture with bacterial hosts. J Oral Microbiol.

[32] Utter DR, He X, Cavanaugh CM, McLean JS, Bor B (2020). The saccharibacterium TM7x elicits differential responses across its host range. ISME J.

[33] Moreira D, Zivanovic Y, López-Archilla AI, Iniesto M, López-García P (2021). Reductive evolution and unique predatory mode in the CPR bacterium Vampirococcus lugosii. Nat Commun.

[34] Giovannoni SJ, Thrash JC, Temperton B (2014). Implications of streamlining theory for microbial ecology. ISME J.

[35] Dufresne A, Garczarek L, Partensky F (2005). Accelerated evolution associated with genome reduction in a free-living prokaryote. Genome Biol.

[36] Küsel K, Totsche KU, Trumbore SE, Lehmann R, Steinhäuser C, Herrmann M (2016). How deep can surface signals be traced in the critical zone? Merging biodiversity with biogeochemistry research in a central German Muschelkalk landscape. Front Earth Sci.

[37] Yan L, Herrmann M, Kampe B, Lehmann R, Totsche KU, Küsel K (2020). Environmental selection shapes the formation of near-surface groundwater microbiomes. Water Res.

[38] Quast C, Pruesse E, Yilmaz P, Gerken J, Schweer T, Yarza P (2012). The SILVA ribosomal RNA gene database project: improved data processing and web-based tools. Nucleic Acids Res.

[39] Eren AM, Esen ÖC, Quince C, Vineis JH, Morrison HG, Sogin ML (2015). Anvi’o: an advanced analysis and visualization platform for ‘omics data. PeerJ.

[40] Eren AM, Kiefl E, Shaiber A, Veseli I, Miller SE, Schechter MS (2021). Community-led, integrated, reproducible multi-omics with anvi’o. Nature Microbiol.

[41] Campbell JH, O’Donoghue P, Campbell AG, Schwientek P, Sczyrba A, Woyke T (2013). UGA is an additional glycine codon in uncultured SR1 bacteria from the human microbiota. Proc Natl Acad Sci.

[42] Kersey CM, Agyemang PA, Dumenyo CK (2012). CorA, the magnesium/nickel/cobalt transporter, affects virulence and extracellular enzyme production in the soft rot pathogen *Pectobacterium carotovorum*. Mol Plant Pathol.

[43] Porcheron G, Garénaux A, Proulx J, Sabri M, Dozois CM (2013). Iron, copper, zinc, and manganese transport and regulation in pathogenic Enterobacteria: correlations between strains, site of infection and the relative importance of the different metal transport systems for virulence. Front Cell Infect Microbiol.

[44] Altschul SF, Gish W, Miller W, Myers EW, Lipman DJ (1990). Basic local alignment search tool. J Mol Biol.

[45] Yan L, Hermans SM, Totsche KU, Lehmann R, Herrmann M, Küsel K (2021). Groundwater bacterial communities evolve over time in response to recharge. Water Res.

[46] Tian R, Ning D, He Z, Zhang P, Spencer SJ, Gao S (2020). Small and mighty: adaptation of superphylum Patescibacteria to groundwater environment drives their genome simplicity. Microbiome.

[47] Castelle CJ, Brown CT, Thomas BC, Williams KH, Banfield JF (2017). Unusual respiratory capacity and nitrogen metabolism in a Parcubacterium (OD1) of the Candidate Phyla Radiation. Sci Rep.

[48] Kohlhepp B, Lehmann R, Seeber P, Küsel K, Trumbore SE, Totsche KU (2017). Aquifer configuration and geostructural links control the groundwater quality in thin-bedded carbonate–siliciclastic alternations of the Hainich CZE, central Germany. Hydrol Earth Syst Sci.

[49] Opitz S, Küsel K, Spott O, Totsche KU, Herrmann M (2014). Oxygen availability and distance to surface environments determine community composition and abundance of ammonia-oxidizing prokaroytes in two superimposed pristine limestone aquifers in the Hainich region, Germany. FEMS Microbiol Ecol.

[50] Wegner C-E, Gaspar M, Geesink P, Herrmann M, Marz M, Küsel K (2019). Biogeochemical regimes in shallow aquifers reflect the metabolic coupling of the elements nitrogen, sulfur, and carbon. Appl Environ Microbiol.

[51] Ozawa K, Meikari T, Motohashi K, Yoshida M, Akutsu H (2000). Evidence for the presence of an F-type ATP synthase involved in sulfate respiration in *Desulfovibrio vulgaris*. J Bacteriol.

[52] Nelson WC, Stegen JC (2015). The reduced genomes of Parcubacteria (OD1) contain signatures of a symbiotic lifestyle. Front Microbiol.

[53] McLean JS, Bor B, Kerns KA, Liu Q, To TT, Solden L (2020). Acquisition and adaptation of ultra-small parasitic reduced genome bacteria to mammalian hosts. Cell Rep.

[54] Batinovic S, Rose JJ, Ratcliffe J, Seviour RJ, Petrovski S (2021). Cocultivation of an ultrasmall environmental parasitic bacterium with lytic ability against bacteria associated with wastewater foams. Nat Microbiol.

[55] Levin PA, Angert ER (2015). Small but mighty: cell size and bacteria. Cold Spring Harb Perspect Biol.

[56] Kempes CP, Wang L, Amend JP, Doyle J, Hoehler T (2016). Evolutionary tradeoffs in cellular composition across diverse bacteria. ISME J.

[57] Melville S, Craig L (2013). Type IV pili in Gram-positive bacteria. Microbiol Mol Biol Rev.

[58] Craig L, Volkmann N, Arvai AS, Pique ME, Yeager M, Egelman EH (2006). Type IV pilus structure by cryo-electron microscopy and crystallography: implications for pilus assembly and functions. Mol Cell.

[59] Mandlik A, Swierczynski A, Das A, Ton-That H (2008). Pili in Gram-positive bacteria: assembly, involvement in colonization and biofilm development. Trends Microbiol.

[60] Xie B, Wang J, Nie Y, Chen D, Hu B, Wu X, et al. EpicPCR-directed cultivation of a candidatus saccharibacteria symbiont reveals a type IV Pili-dependent epibiotic lifestyle. bioRxiv. https://www.biorxiv.org/content/early/2021/07/08/2021.07.08.451036.

[61] Coleman GA, Davín AA, Mahendrarajah TA, Szánthó LL, Spang A, Hugenholtz P (2021). A rooted phylogeny resolves early bacterial evolution. Science.

[62] Lehmann K, Lehmann R, Totsche KU (2021). Event-driven dynamics of the total mobile inventory in undisturbed soil account for significant fluxes of particulate organic carbon. Sci Total Environ.

[63] Nurk S, Meleshko D, Korobeynikov A, Pevzner PA (2017). metaSPAdes: a new versatile metagenomic assembler. Genome Res.

[64] Ultsch A, Mörchen F (2005). ESOM-Maps: tools for clustering, visualization, and classification with Emergent SOM.

[65] Dick GJ, Andersson AF, Baker BJ, Simmons SL, Thomas BC, Yelton AP (2009). Community-wide analysis of microbial genome sequence signatures. Genome Biol.

[66] Wu Y-W, Simmons BA, Singer SW (2016). MaxBin 2.0: an automated binning algorithm to recover genomes from multiple metagenomic datasets. Bioinformatics.

[67] Parks DH, Imelfort M, Skennerton CT, Hugenholtz P, Tyson GW (2015). CheckM: assessing the quality of microbial genomes recovered from isolates, single cells, and metagenomes. Genome Res.

[68] Olm MR, Brown CT, Brooks B, Banfield JF (2017). dRep: a tool for fast and accurate genomic comparisons that enables improved genome recovery from metagenomes through de-replication. ISME J.

[69] Gruber-Vodicka HR, Seah BK, Pruesse E (2020). phyloFlash: rapid small-subunit rRNA profiling and targeted assembly from metagenomes. Msystems.

[70] Chaumeil P-A, Mussig AJ, Hugenholtz P, Parks DH (2020). GTDB-Tk: a toolkit to classify genomes with the genome taxonomy database.

[71] Hyatt D, Chen G-L, LoCascio PF, Land ML, Larimer FW, Hauser LJ (2010). Prodigal: prokaryotic gene recognition and translation initiation site identification. BMC Bioinform.

[72] Shaffer M, Borton MA, McGivern BB, Zayed AA, La Rosa SL, Solden LM (2020). DRAM for distilling microbial metabolism to automate the curation of microbiome function. Nucleic Acids Res.

[73] Kanehisa M, Furumichi M, Tanabe M, Sato Y, Morishima K (2017). KEGG: new perspectives on genomes, pathways, diseases and drugs. Nucleic Acids Res.

[74] Rawlings ND, Barrett AJ, Bateman A (2010). MEROPS: the peptidase database. Nucleic Acids Res.

[75] El-Gebali S, Mistry J, Bateman A, Eddy SR, Luciani A, Potter SC (2019). The Pfam protein families database in 2019. Nucleic Acids Res.

[76] Zhang H, Yohe T, Huang L, Entwistle S, Wu P, Yang Z (2018). dbCAN2: a meta server for automated carbohydrate-active enzyme annotation. Nucleic Acids Res.

[77] Edgar RC (2004). MUSCLE: multiple sequence alignment with high accuracy and high throughput. Nucleic Acids Res.

[78] Price MN, Dehal PS, Arkin AP (2010). FastTree 2-approximately maximum-likelihood trees for large alignments. PLoS ONE.

[79] Emiola A, Oh J (2018). High throughput in situ metagenomic measurement of bacterial replication at ultra-low sequencing coverage. Nat Commun.

[80] R Core Team. R: a language and environment for statistical computing. R Foundation for Statistical Computing; 2020. https://www.R-project.org/.

[81] Quinn TP, Richardson MF, Lovell D, Crowley TM (2017). propr: an R-package for identifying proportionally abundant features using compositional data analysis. Sci Rep.

[82] Csardi G, Nepusz T (2006). The igraph software package for complex network research. InterJournal Complex Syst.

[83] Shannon P, Markiel A, Ozier O, Baliga NS, Wang JT, Ramage D (2003). Cytoscape: a software environment for integrated models of biomolecular interaction networks. Genome Res.

[84] Gustavsen JA, Pai S, Isserlin R, Demchak B, Pico AR (2019). RCy3: network biology using Cytoscape from within R. F1000Research.

[85] Kassambara A. rstatix: pipe-friendly framework for basic statistical tests. 2020. https://CRAN.R-project.org/package=rstatix.

