# Supplementary figures and images for: The economical lifestyle of CPR bacteria in groundwater allows little preference for environmental drivers
Source: Environ Microbiome. 2021 Dec 14;16:24. doi: 10.1186/s40793-021-00395-w (PMC8672522; doi:10.1186/s40793-021-00395-w)

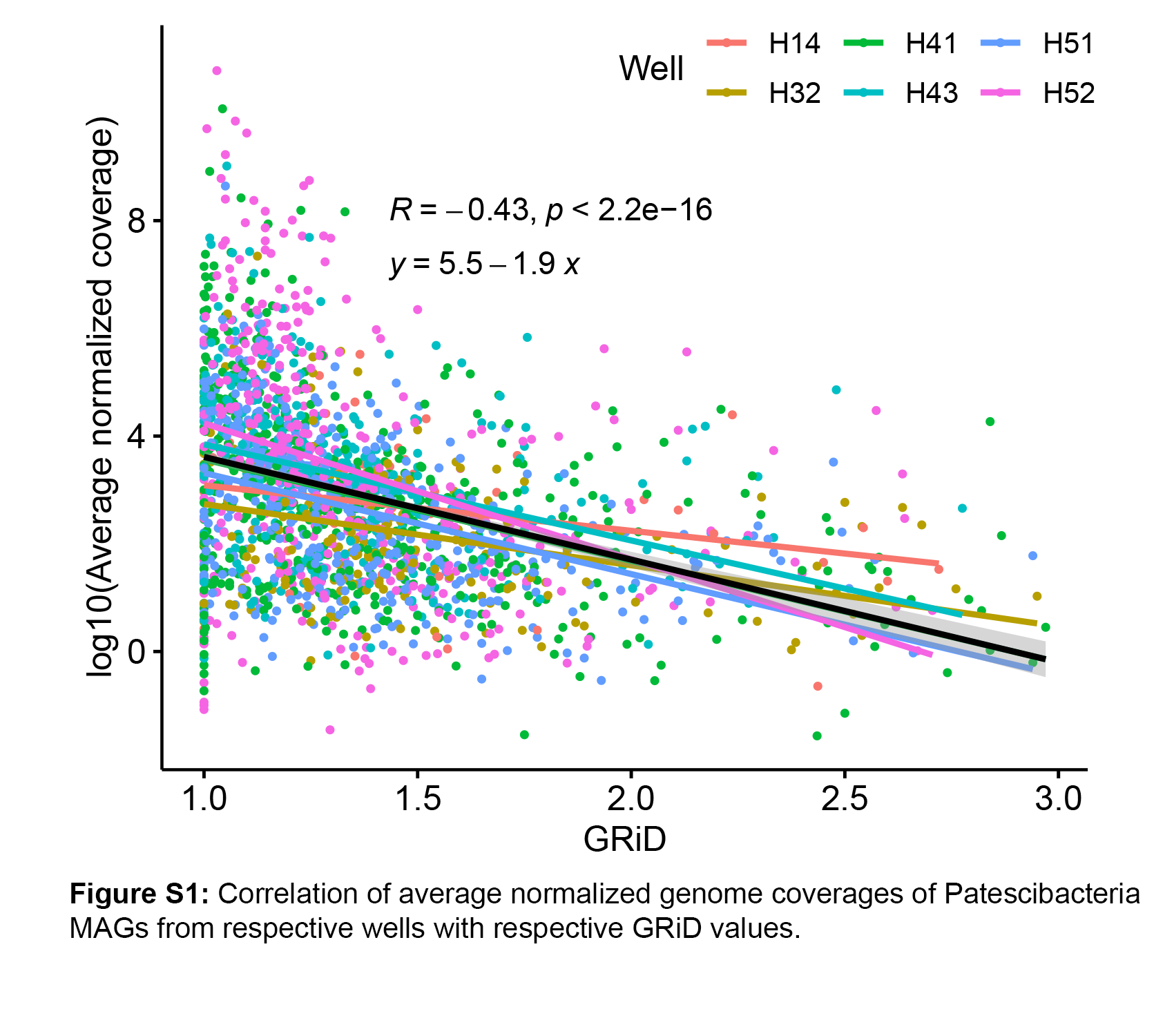

Supplement: Supplementary file 4 — Additional file 4: Fig. S1. Correlation of average normalized genome coverages of Patescibacteria MAGs from respective wells with respective GRiD values. [file 40793_2021_395_MOESM4_ESM.tif]

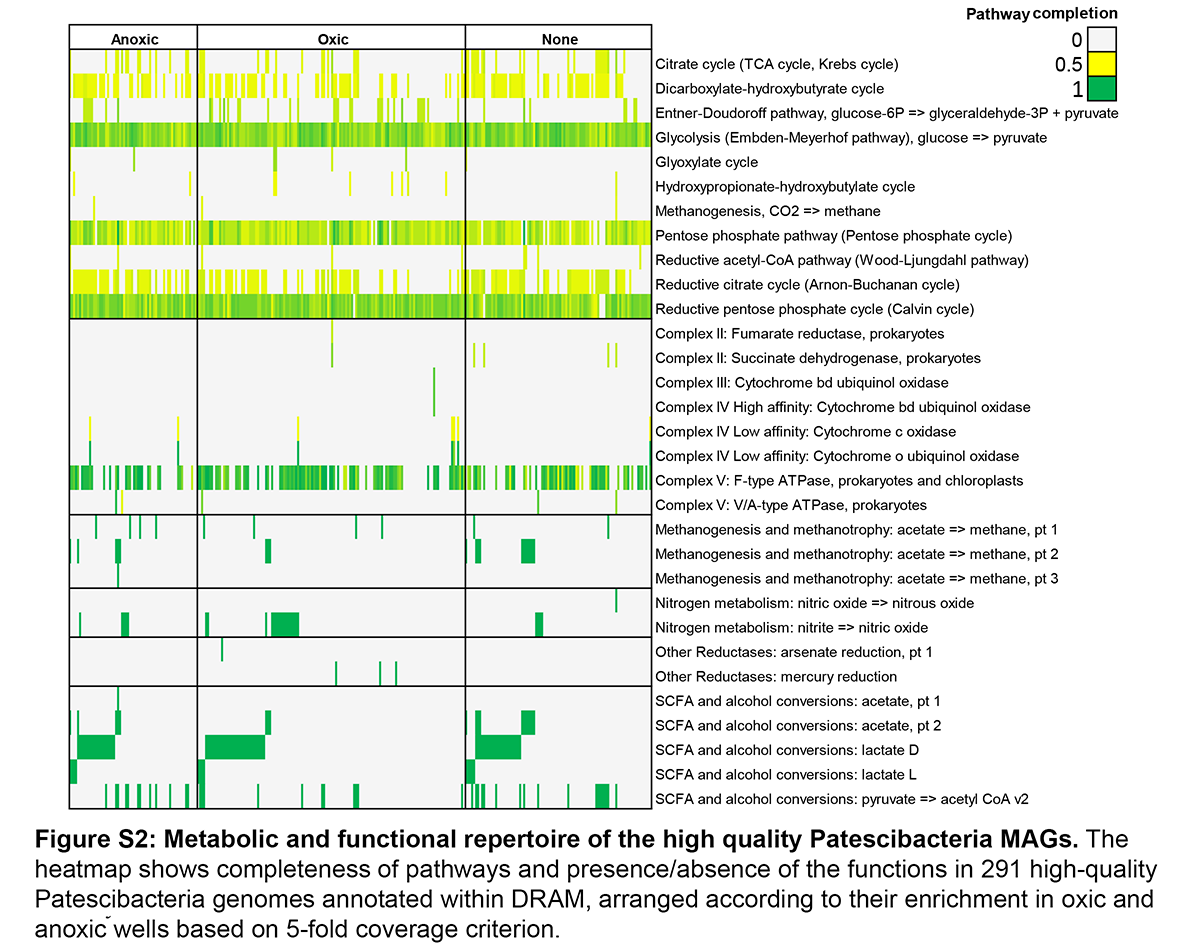

Supplement: Supplementary file 5 — Additional file 5: Fig. S2. Metabolic and functional repertoire of high quality Patescibacteria MAGs. The heatmap shows completeness of pathways and presence/absence of functions in 291 high-quality Patescibacteria genomes annotated with DRAM, arranged according to their enrichment in oxic and anoxic wells based on 5-fold coverage criterion. [file 40793_2021_395_MOESM5_ESM.tif]

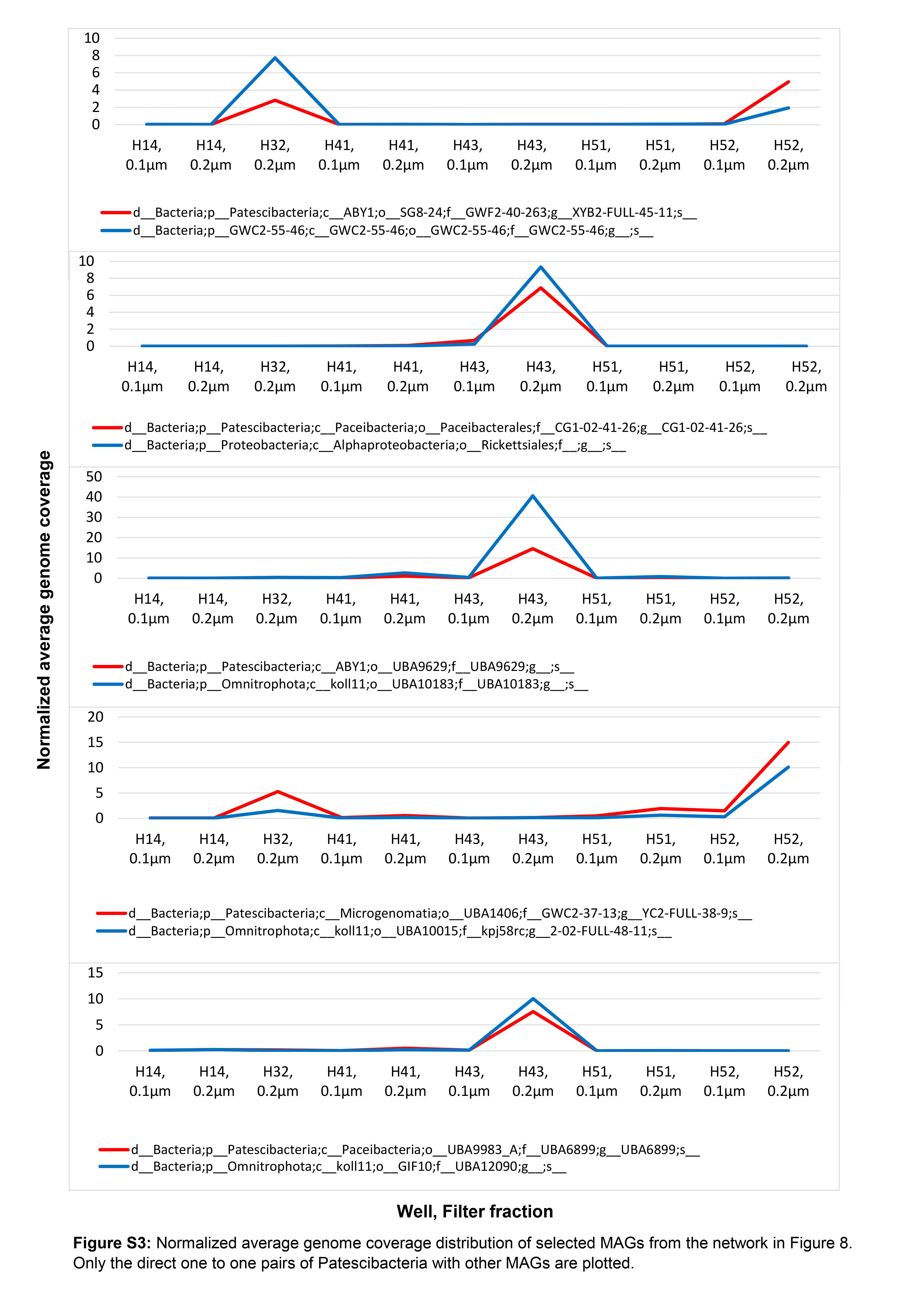

Supplement: Supplementary file 8 — Additional file 8: Fig. S3. Coverage distribution of selected MAGs from the network in Fig. 8. Only the direct one-to-one pairs of Patescibacteria with other MAGs are plotted. [file 40793_2021_395_MOESM8_ESM.tif]

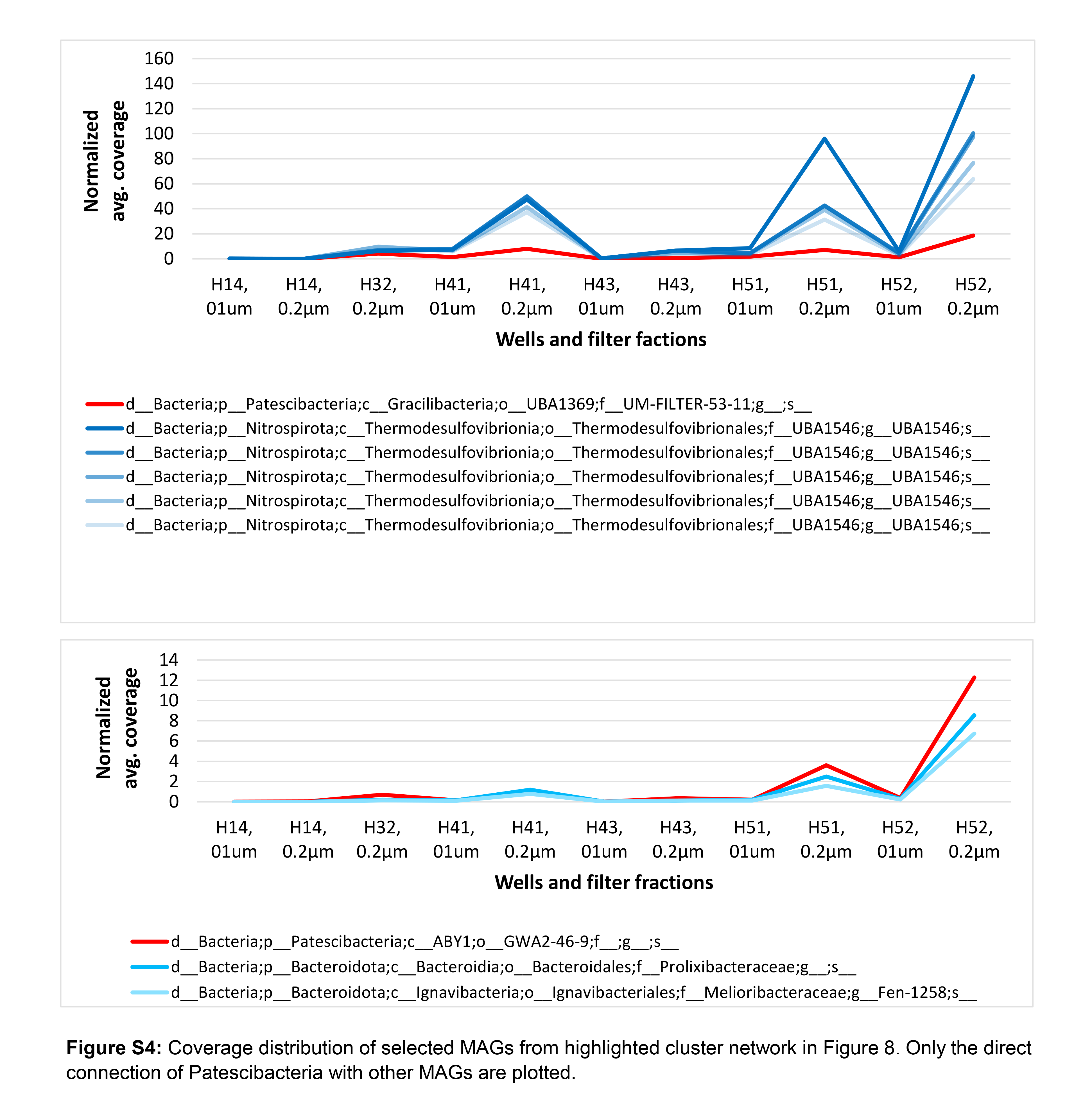

Supplement: Supplementary file 9 — Additional file 9: Fig. S4. Coverage distribution of selected MAGs from the highlighted cluster in network in Fig. 8. Only the direct connections of Patescibacteria with other MAGs are plotted. [file 40793_2021_395_MOESM9_ESM.tif]
